# Associations of Habitual Mineral Intake with New-Onset Prediabetes/Diabetes after Acute Pancreatitis

**DOI:** 10.3390/nu13113978

**Published:** 2021-11-08

**Authors:** Claire F. Norbitt, Wandia Kimita, Juyeon Ko, Sakina H. Bharmal, Maxim S. Petrov

**Affiliations:** School of Medicine, University of Auckland, Auckland 1023, New Zealand; cnor327@aucklanduni.ac.nz (C.F.N.); wandia.kimita@auckland.ac.nz (W.K.); ju.ko@auckland.ac.nz (J.K.); s.bharmal@auckland.ac.nz (S.H.B.)

**Keywords:** manganese, iron, iodine, selenium, habitual mineral intake, pancreatitis, diabetes, glucose metabolism, insulin traits

## Abstract

Associations between habitual dietary intake of minerals and glucose metabolism have been extensively studied in relation to metabolic disorders. However, similar research has yet to be conducted in individuals after acute pancreatitis (AP). The main aim was to investigate the associations between habitual intake of 13 minerals and glycaemic status: new-onset prediabetes/diabetes after AP (NODAP), pre-existing prediabetes/type 2 diabetes (T2DM), and normoglycaemia after AP (NAP). Associations between the dietary intake of minerals and markers of glucose metabolism (glycated haemoglobin and fasting plasma glucose) were also studied. The EPIC-Norfolk food frequency questionnaire was used in a cross-sectional fashion to determine the habitual intake of 13 dietary minerals. ANCOVA as well as multiple linear regression analyses were conducted and five statistical models were built to adjust for covariates. The study included 106 individuals after AP. In the NODAP group, intake of 4 minerals was significantly less when compared with the NAP group: iron (B = −0.076, *p* = 0.013), nitrogen (B = −0.066, *p* = 0.003), phosphorous (B = −0.046, *p* = 0.006), and zinc (B = −0.078, *p* = 0.001). Glycated haemoglobin was significantly associated with iodine intake (B = 17.763, *p* = 0.032) and manganese intake (B = −17.147, *p* = 0.003) in the NODAP group. Fasting plasma glucose was significantly associated with manganese intake (B = −2.436, *p* = 0.027) in the NODAP group. Habitual intake of minerals differs between individuals with NODAP, T2DM, and NAP. Prospective longitudinal studies and randomised controlled trials are now warranted to further investigate the associations between mineral intake and NODAP.

## 1. Introduction

Diabetes mellitus is the most frequent non-communicable disease worldwide. Classifications of diabetes include both the widely recognised type 1 and type 2 and less appreciated types of secondary diabetes, such as diabetes of the exocrine pancreas (DEP) [1,2]. New-onset diabetes after acute pancreatitis is the most frequent DEP, characterised by dysfunction of endocrine cells in the pancreas secondary to an attack of acute pancreatitis (AP) [3,4,5]. The risk of new-onset diabetes after acute pancreatitis is at least 2 times higher in individuals after an attack of AP compared with the general population [6,7]. Also, a prospective longitudinal cohort study by the COSMOS group observed glucose metabolism derangement occurred progressively after an attack of AP, with 11% of individuals (who did not have diabetes at the time of hospitalisation) developing new-onset diabetes after acute pancreatitis up to 24 months post AP attack [8]. Further studies have found the incidence of AP and new-onset diabetes after acute pancreatitis rising over the years; consequently, the burden of these diseases is also increasing and is expected to keep rising over the next three decades [2,4]. New-onset diabetes after acute pancreatitis is commonly unrecognised and misdiagnosed as type 2 diabetes; however, there are marked differences between them. People with new-onset diabetes after acute pancreatitis have poorer glycaemic control, increased risk of developing cancer (in particular, pancreatic cancer), have a younger average age at death, and increased risk of mortality [1,5,9]. Men [7,10], young to middle-aged adults [11], and lean or overweight individuals [5] are also at higher risk of new-onset diabetes after acute pancreatitis compared with type 2 diabetes. Due to these established differences between the types of diabetes, treating new-onset diabetes after acute pancreatitis as type 2 diabetes is detrimental to optimal management [1,5,12].

Current first-line prevention and non-pharmaceutical management of type 2 diabetes is nutrition therapy—an integral component of a diabetes treatment plan [13,14]. Nutrition therapy improves glycaemic control, insulin resistance, and also aids weight loss, resulting in decreased mortality and morbidity associated with type 2 diabetes [13,15,16,17]. Individualised nutrition therapy includes modifying patients’ dietary intake, moving towards a healthful diet prioritising whole foods while reducing intake of processed, less nutritious, and energy-dense foods [13]. Current nutritional research for treatment of type 2 diabetes predominantly focuses on altered dietary intake, dietary patterns, and macronutrient intake, with less focus on micronutrient intake [16]. At the same time, there are no current disease specific nutrition interventions for those at risk of, or with new-onset diabetes after acute pancreatitis, with these people receiving generalised nutrition advice. Minerals are essential for glucose metabolism by serving as co-factors, activating insulin receptor sites, and affecting insulin sensitivity [18,19]. Previous studies investigating the role of minerals in type 2 diabetes observed that dietary intake of calcium [18,20], magnesium [20,21,22], and manganese [23,24,25] may have protective effects on type 2 diabetes, while increased dietary iron [26,27,28,29,30,31] and selenium [32,33,34] intake may increase risk of this type of diabetes. To the best of our knowledge, similar research has yet to be conducted on associations of habitual mineral intake with new-onset diabetes after acute pancreatitis. We hypothesised that habitual mineral intake plays a role in the dysregulation of glucose metabolism after AP.

The present study primarily aimed to investigate the associations between habitual intake of minerals and glycaemic status after AP. Secondary aims were to assess associations between the dietary intake of minerals and markers of glucose metabolism (fasting plasma glucose (FPG) and glycated haemoglobin (HbA1c)), and insulin traits (fasting insulin, homeostasis model assessment insulin sensitivity (HOMA-S) index, and homeostasis model assessment β-cell function (HOMA-β) index).

## 2. Methods

### 2.1. Study Design

This cross-sectional study investigated individuals after an attack of AP as part of the ANDROMEDA (Assessment of Nutritional and DietaRy factOrs in Metabolic Disorders after pAncreatitis) project conducted by the COSMOS group.

### 2.2. Study Population

Individuals were eligible to participate in the study if they fulfilled the following inclusion criteria: primary diagnosis of AP between 2015–2019, at least 18 years of age, reside in Auckland at the time of the study, and provided informed consent for participation. Diagnosis of AP was in line with the most up-to-date international guidelines, requiring at least two of the three following criteria to be present: abdominal pain suggestive of AP (acute onset of persistent and severe epigastric pain, often radiating to the back), elevated serum lipase and/or amylase levels at least three times greater than the upper limit of normal, and/or findings characteristic of AP in contrast-enhanced computed tomography, magnetic resonance imaging (MRI), or ultrasound studies [35]. Individuals were excluded if they met any of the following criteria: diagnosis of chronic pancreatitis, intraoperative diagnosis of pancreatitis, post-endoscopic retrograde cholangiopancreatography pancreatitis, history of type 1 or gestational diabetes, pregnancy during AP or hereafter, malignancy, history of steroid use, coeliac disease, or cystic fibrosis.

### 2.3. Study Groups

Study participants were categorised into three groups based on their HbA1c and FPG levels at the time of the study, in line with the ‘DEP criteria’ [36]. Those with HbA1c < 5.7% (39 mmol/mol) and/or FPG < 100 mg/dL (5.6 mmol/L) both at the time of their qualifying attack of AP and at the time of the study and made up the normoglycaemia after AP (NAP) group. Participants with type 2 diabetes or prediabetes (T2DM) before their qualifying attack of AP had an HbA1c ≥ 5.7% (39 mmol/mol) and/or FPG ≥ 100 mg/dL (5.6 mmol/L) at the time of the study made up the T2DM group. Last, those who had normoglycaemia before and during their qualifying AP attack but had HbA1c ≥ 5.7% (39 mmol/mol) and/or FPG ≥ 100 mg/dL (5.6 mmol/L) at follow-up made up the new-onset prediabetes/diabetes after pancreatitis (NODAP) group. Participants who had FPG >100 mg/dL (5.6 mmol/L) but HbA1c < 5.7% (39 mmol/mol) during their qualifying AP attack were not considered to account for effects of transient stress hyperglycaemia [37].

### 2.4. Ascertainment of Mineral Intake

Participants’ habitual diet over the year before recruitment was assessed using the EPIC-Norfolk food frequency questionnaire (FFQ) developed by the University of Cambridge [38]. The FFQ is a self-administered, validated, and semi-quantitative instrument that consists of two parts. Part one assesses the intake of 130 commonly and less commonly consumed foods. Part two consists of additional questions, gathering information on types and brands of foods such as breakfast cereal, milk, meat, and cooking fats. Ascertainment of habitual intake included minerals from food sources only; therefore, intake of supplements was not considered in the present study. The FFQ data were analysed using FETA (FFQ EPIC Tool for Analysis) software to calculate daily intake of 13 minerals: calcium (mg), chloride (mg), copper (mg), iodine (µg), iron (mg), magnesium (mg), manganese (mg), nitrogen (g), phosphorous (mg), potassium (mg), selenium (µg), sodium (mg), and zinc (mg). FFQs were excluded from the study if ten or more questions were left unanswered as this level of missing data would lead to significant underestimation of intake [38]. In addition, FFQ data were excluded if the ratio of total energy intake (estimated from the FFQ data) and estimated basal metabolic rate (determined by the Harris-Benedict equation) were more than two standard deviations (SD) outside the mean ratio (i.e., <0.28 and >1.82) [38].

### 2.5. Laboratory Assays

Venous blood samples were collected from participants to measure HbA1c (mmol/mol), FPG (mmol/L), and fasting insulin (mU/L). All participants were required to fast 8 h before blood collection to establish a fasted blood sample. Lab Plus (International Accreditation New Zealand accredited medical laboratory at Auckland City Hospital) analysed fresh samples. A Homeostasis Model Assessment calculator (HOMA2), developed by Oxford University, was used to estimate HOMA-S and HOMA- β indices as percentages of a normal reference population (version 2.2.4 Diabetes Trials Unit, University of Oxford, Oxford, UK).

### 2.6. Covariates

Demographic data, including age and sex, were collected during a standardised face-to-face health examination conducted by the COSMOS team. Participants undertook abdominal magnetic resonance imaging at the Centre for Advanced Magnetic Resonance Imaging (University of Auckland, Auckland, New Zealand) in order to measure abdominal visceral fat volume (VFV), subcutaneous fat volume (SFV) and, subsequently, visceral to subcutaneous fat volume ratio (V/S fat volume ratio). A 3T MAGNETOM Skyra scanner (Siemens, Erlangen, Germany) was used. Participants were asked to lie supine and hold their breath at the end of expiration. Axial T1-weighted volumetric interpolated breath-hold examination Dixon sequence was applied as reported elsewhere [39]. Visceral and subcutaneous fat volume was quantified using ImageJ software (National Institutes of Health, Bethesda, MD, USA). Abdominal fat phase images from the second lumbar vertebral level (L2) to the fifth lumbar vertebral level (L5) were used to measure subcutaneous and visceral fat depots [40]. The threshold function of ImageJ was used to convert grayscale pixels into binary images using the global histogram-derived method [39]. The non-adipose tissue was excluded from the measurement of visceral fat. The total number of pixels from the slices series was calculated and multiplied by the pixel area and slice thickness to obtain the VFV and SFV [41]. Subsequently, the ratio of V/S fat volume ratio was calculated.

Energy intake was defined as the average daily intake of calories (kcal) from food consumption assessed using the FFQ and determined by the FETA software, as was daily alcohol intake (g/day) [38]. Tobacco smoking status was established at the time of the MRI scan using a standardised questionnaire [42]. Smoking status was categorised into never, former, light (<20 cigarettes/day), moderate (20–39 cigarettes/day) and heavy (>40 cigarettes/day). Antidiabetic medications and cholecystectomy data were derived from participants’ health records on Concerto (Concerto ^TM^ software, Orion Health Group Ltd., Auckland, New Zealand). Information on the aetiology of AP was also acquired from health records and was categorised into biliary, alcohol-related, and other.

### 2.7. Statistical Analysis

All statistical analyses were performed using SPSS 27.0. (IBM Corporation, Armonk, NY, USA). The differences in baseline characteristics between the study groups (NODAP, T2DM, and NAP) were investigated using one-way ANOVA. Data were presented as mean (standard deviation) or frequency (percentage). First, analysis of covariance (ANCOVA) between the NODAP, T2DM, and NAP groups (reference group) was undertaken to assess variance in mean mineral intakes between the groups while adjusting for the effect of covariates. All investigated minerals were log-transformed to account for non-normal distribution (based on the Shapiro-Wilk test). Five models were built for ANCOVA analysis. Model 1 was unadjusted; model 2 was adjusted for age, sex, and daily energy intake; model 3 was adjusted for age, sex, daily energy intake, and V/S fat volume ratio; model 4 was adjusted for age, sex, daily energy intake, V/S fat volume ratio, smoking status, and daily alcohol intake; model 5 was adjusted for age, sex, daily energy intake, V/S fat volume ratio, smoking status, daily alcohol intake, aetiology of AP, number of AP episodes, cholecystectomy, and use of antidiabetic medications. Data were presented as a β coefficient, *p* value, and 95% confidence interval. Last, to investigate the associations between the investigated minerals and markers of glucose metabolism as well as insulin traits, multiple linear regression analyses were conducted for each study group. Each marker of glucose metabolism and insulin trait was treated as a dependent variable. Multiple linear regression analyses were conducted using the same five statistical models as the ANCOVA analysis. Data were presented as R^2^, unstandardised B, *p* value, and 95% confidence interval. *P* values less than 0.05 were considered statistically significant in all analyses, and data were not corrected for multiple tests.

## 3. Results

### 3.1. Characteristics of the Study Cohort

A total of 106 eligible individuals diagnosed with AP were included in the present study. The mean and standard deviation time since the last AP attack was 26 ± 20 months, and the number of participants with recurrent attacks of AP did not differ significantly between the groups (*p* = 0.125). The NODAP group consisted of 37 participants, the T2DM group consisted of 37 participants, and the NAP group consisted of 32 participants. Table 1 shows the characteristics of the study cohort. There were statistically significant differences in means between the three groups for the following characteristics: V/S fat volume ratio (*p* = 0.035), use of antidiabetic medications (*p* < 0.001), HbA1c (mmol/mol) (*p* < 0.001), and FPG (mmol/L) (*p* < 0.001).

### 3.2. Associations between Habitual Mineral Intake and Diabetes Types

In the NODAP group, four minerals (iron, nitrogen, phosphorous, zinc) were significantly different when compared with the NAP group (Table 2). The mean iron intake was significantly different in all adjusted models (*p* = 0.029 in model 2, *p* = 0.030 in model 3, *p* = 0.036 in model 4, and *p* = 0.013 in model 5). The mean nitrogen intake was significantly different in all adjusted models (*p* = 0.003 in model 2, *p* = 0.003 in model 3, *p* = 0.002 in model 4, and *p* = 0.003 in model 5). The mean phosphorous intake was significantly different in all adjusted models (*p* = 0.005 in model 2, *p* = 0.005 in model 3, *p* = 0.005 in model 4, and *p* = 0.006 in model 5). The mean zinc intake was significantly different in all adjusted models (*p* = 0.002 in model 2, *p* = 0.003 in model 3, *p* = 0.003 in model 4, and *p* = 0.001 in model 5). Intake of other investigated minerals in the NODAP group did not differ significantly from the reference group.

In the T2DM group, one mineral was significantly different when compared with the NAP group (Table 2). The mean copper intake was significantly different in the unadjusted model (*p* = 0.012) and adjusted models 2–4 (*p* = 0.039 in model 2, *p* = 0.026 in model 3, and *p* = 0.049 in model 4). However, the difference in mean intake became insignificant in the most adjusted model (*p* = 0.147). Intake of other investigated minerals in the T2DM group did not differ significantly from the reference group.

### 3.3. Associations between Habitual Mineral Intake and Markers of Glucose Metabolism in the Study Groups

**Figure 1:**
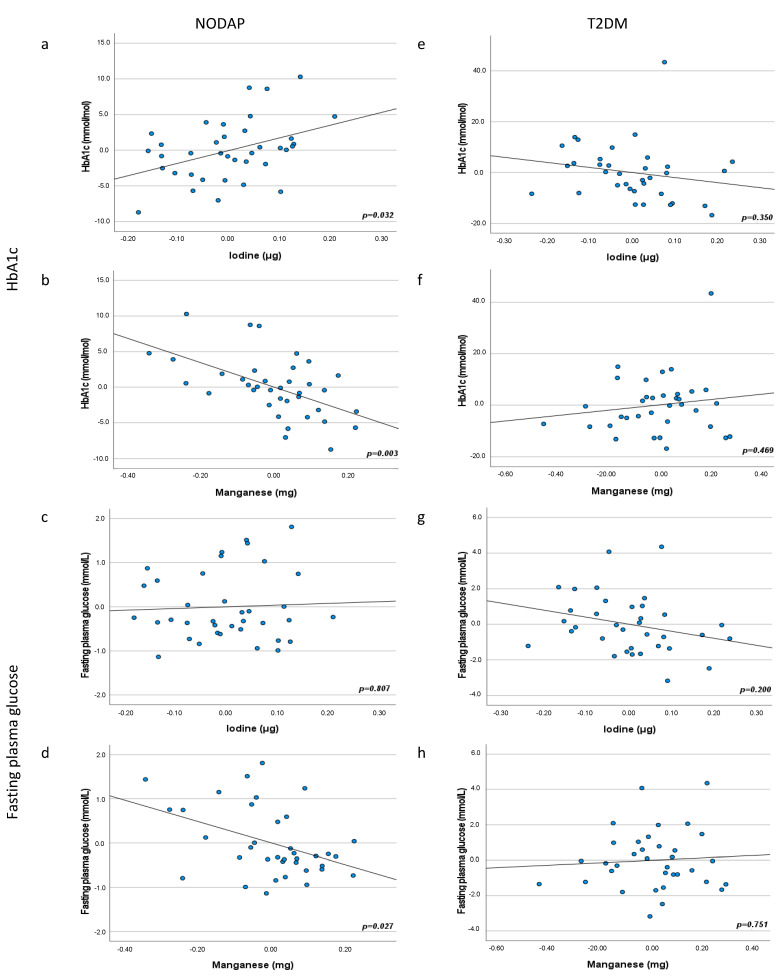
Associations between iodine and manganese intake and markers of glucose metabolism in NODAP (**a–d**) and T2DM (**e–h**). Abbreviations: NODAP = New-onset diabetes or prediabetes after acute pancreatitis. T2DM = Type 2 diabetes or prediabetes prior to acute pancreatitis. HbA1c = glycated haemoglobin. Iodine and manganese data were log transformed. Partial regression plots were adjusted for age, sex, daily energy intake, V/S fat volume, alcohol intake, smoking status, aetiology of AP, number of AP episodes, cholecystectomy, use of antidiabetic medications. Significance was set at *p* < 0.05.

HbA1c levels were associated with two minerals in the NODAP group. Iodine intake was significantly directly associated with HbA1c levels in adjusted models 3 and 5 (*p* = 0.037 and *p* = 0.032, respectively) (Table 3, Figure 1). Manganese intake was significantly inversely associated with HbA1c in the NODAP group in all adjusted models (*p* = 0.003 in model 2, *p* = 0.003 in model 3, *p* = 0.002 in model 4, and *p* = 0.003 in model 5) (Figure 1). Associations between intake of other investigated minerals and HbA1c in the NODAP group were not statistically significant.

In the T2DM group and the NAP group, there were no statistically significant associations between any investigated minerals and HbA1c levels in all models.

FPG levels were associated with one mineral in the NODAP group (Table 4, Figure 1). Manganese was significantly inversely associated with FPG in all adjusted models (*p* = 0.029 in model 2, *p* = 0.031 in model 3, *p* = 0.020 in model 4, and *p* = 0.027 in model 5) (Figure 1).

In the T2DM group, associations between the investigated minerals and FPG were not statistically significant (Table 4).

In the NAP group, FPG levels were associated with three minerals (copper, magnesium, and potassium) (Table 4). Copper intake was significantly inversely associated with FPG levels in adjusted models 2 and 5 (*p* = 0.044 in model 2 and *p* = 0.023 in model 5). Magnesium intake was significantly inversely associated with FPG levels in all adjusted models (*p* = 0.008 in model 2, *p* = 0.023 in model 3, *p* = 0.027 in model 4, and *p* = 0.030 in model 5). Potassium intake was significantly inversely associated with FPG levels in all adjusted models (*p* = 0.011 in model 2, *p* = 0.029 in model 3, *p* = 0.031 in model 4, and *p* = 0.036 in model 5).

### 3.4. Associations between Habitual Mineral Intake and Insulin Traits in the Study Groups

Fasting insulin levels were associated with two minerals (chloride and sodium) in the NODAP group (Table 5). Chloride intake was significantly directly associated with fasting insulin levels in the unadjusted model only (*p* = 0.044). Sodium was significantly directly associated with fasting insulin levels in the unadjusted model only (*p* = 0.043).

**Figure 2:**
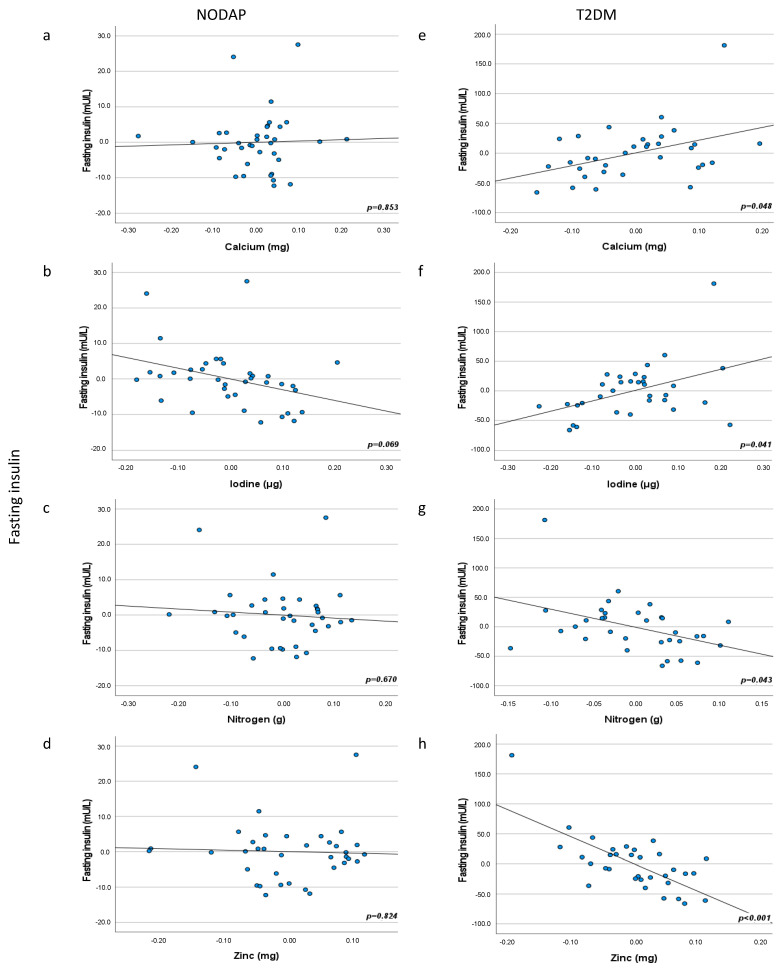
Associations between calcium, iodine, nitrogen, and zinc intake and fasting insulin in NODAP (**a**–**d**) and T2DM (**e**–**h**). Abbreviations: NODAP = New-onset diabetes or prediabetes after acute pancreatitis. T2DM = Type 2 diabetes or prediabetes prior to acute pancreatitis. HbA1c = glycated haemoglobin. Calcium, iodine, nitrogen, and zinc data were log transformed. Partial regression plots were adjusted for age, sex, daily energy intake, V/S fat volume, alcohol intake, smoking status, aetiology of AP, number of AP episodes, cholecystectomy, use of antidiabetic medications. Significance was set at *p* < 0.05.

Fasting insulin was associated with seven minerals (calcium, chloride, iodine, iron, nitrogen, sodium, and zinc) in the T2DM group (Table 5). Calcium intake was inversely associated with fasting insulin levels in the most adjusted model (*p* = 0.048) (Figure 2). Chloride intake was significantly inversely associated with fasting insulin in the unadjusted model (*p* = 0.043) and adjusted models 2 and 3 (*p* = 0.035 in model 2 and *p* = 0.039 in model 3). Iodine intake was significantly associated in the most adjusted models 4 and 5 (*p* = 0.042 in model 4 and *p* = 0.041 in model 5) (Figure 2). Iron intake was inversely associated with fasting insulin levels in adjusted model 4 only (*p* = 0.028). Nitrogen intake was significantly inversely associated with fasting insulin in both the unadjusted model (*p* = 0.032) and all adjusted models (*p* = 0.026 in model 2, *p* = 0.028 in model 3, *p* = 0.010 in model 4, and *p* = 0.043 in model 5) (Figure 2). Sodium intake was significantly inversely associated with fasting insulin in the unadjusted model (*p* = 0.022) and adjusted models 2 and 3 (*p* = 0.008 in model 2 and *p* = 0.010 in model 3). Zinc intake was significantly inversely associated with fasting insulin levels in both the unadjusted model (*p* = 0.007) and all adjusted models (*p* = 0.001 in model 2, *p* = 0.001 in model 3, *p* < 0.001 in model 4, and *p* < 0.001 in model 5) (Figure 2).

Fasting insulin was not significantly associated with the investigated minerals in the NAP group.

**Figure 3:**
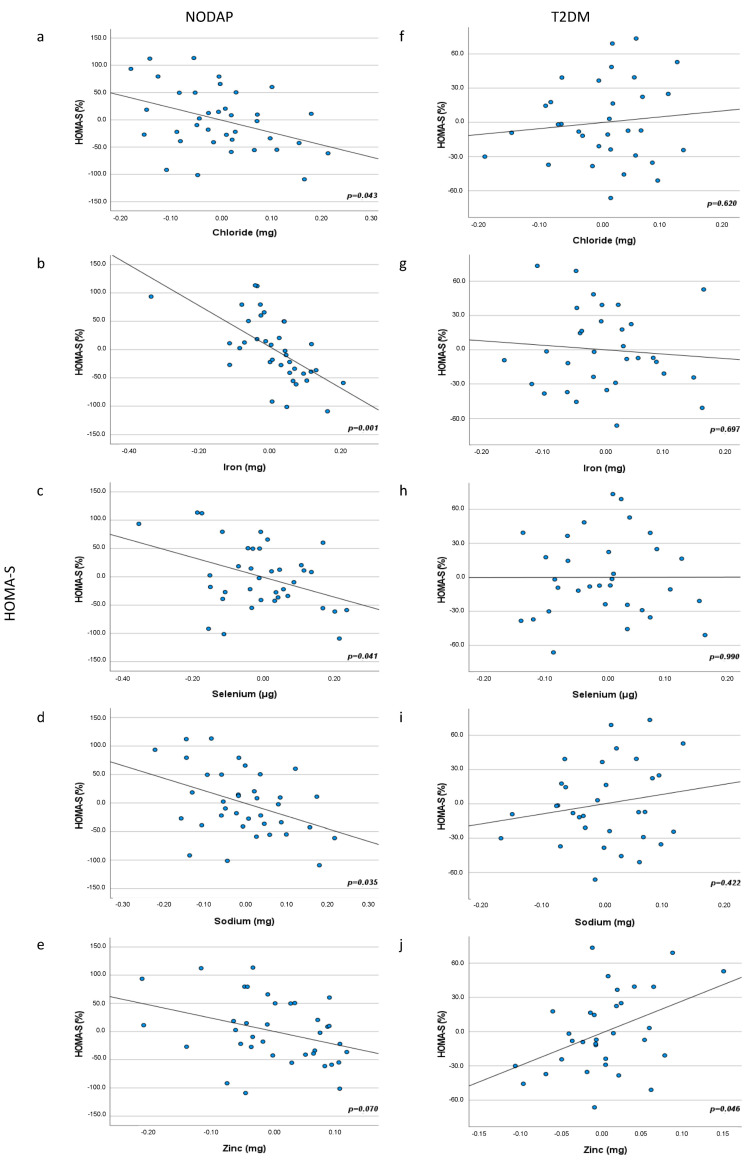
Associations between chloride, iron, selenium, sodium, and zinc intake and HOMA-S in NODAP (**a–e**) and T2DM (**f–j**). Abbreviations: NODAP = New-onset diabetes or prediabetes after acute pancreatitis. T2DM = Type 2 diabetes or prediabetes prior to acute pancreatitis. HbA1c = glycated haemoglobin. Chloride, iron, selenium, sodium, and zinc data were log transformed. Partial regression plots were adjusted for age, sex, daily energy intake, V/S fat volume, alcohol intake, smoking status, aetiology of AP, number of AP episodes, cholecystectomy, use of antidiabetic medications. Significance was set at *p* < 0.05.

HOMA-S was associated with four minerals (chloride, iron, selenium, sodium) in the NODAP group (Table 6). Chloride intake was significantly inversely associated with HOMA-S in the unadjusted model (*p* = 0.044) and the most adjusted model (*p* = 0.044) (Figure 3). Iron intake was significantly inversely associated with HOMA-S in the unadjusted model (*p* = 0.040) and adjusted models (*p* = 0.020 in model 4 and *p* = 0.001 in model 5) (Figure 3). Selenium intake was significantly inversely associated with HOMA-S in both the unadjusted (*p* = 0.015) and all adjusted models (*p* = 0.010 in model 2, *p* = 0.010 in model 3, *p* = 0.014 in model 4, and *p* = 0.042 in model 5) (Figure 3). Sodium intake was significantly inversely associated with HOMA-S in the unadjusted model (*p* = 0.033) and the most adjusted model (*p* = 0.035) (Figure 3).

HOMA-S was associated with one mineral in the T2DM group (Table 6). Zinc intake was significantly directly associated with HOMA-S in two adjusted models (*p* = 0.023 in model 4 and *p* = 0.037 in model 5) (Figure 3).

HOMA-S was not significantly associated with any of the investigated minerals in the NAP group.

HOMA-β was associated with one mineral in the NODAP group (Table 7). Magnesium was significantly directly associated with HOMA-β in the most adjusted models (*p* = 0.035). HOMA-β was not significantly associated with any of the investigated minerals in the T2DM or NAP group (Table 7).

## 4. Discussion

The present study was the first to investigate habitual mineral intake in people with NODAP. The study compared the mean habitual intake of 13 minerals between the NODAP and NAP groups and assessed associations between habitual mineral intake and markers of glucose metabolism, as well as insulin traits, in these groups. A key finding was significant associations between iron, nitrogen, phosphorous, and zinc intakes and the NODAP group, but not the T2DM group. Another key finding was significant associations between manganese, iodine, and markers of glucose metabolism in the NODAP group. Specifically, a significant inverse relationship was observed between manganese intake and both HbA1c and FPG, whereas iodine was significantly directly related to HbA1c levels. Five minerals were also significantly associated with insulin traits in the NODAP group. Specifically, magnesium intake was directly associated with HOMA-β whereas chloride, iron, selenium, and sodium intakes were significantly inversely associated with HOMA-S in people with NODAP.

### 4.1. Manganese Intake and Glucose Metabolism

Manganese is an essential trace element primarily obtained through the dietary intake of grain and cereal products, vegetables, and beverages (tea) [43]. Absorption of manganese is limited, with only 1–5% of ingested intake being absorbed through the small intestine [44,45]. Once absorbed, manganese is transported to mitochondria-rich organs (such as the liver, pituitary gland, and pancreas) [44,45]. Manganese is involved in many processes throughout the body, including enzyme synthesis and activation, metabolism of glucose and lipids, haematopoiesis, endocrine regulation, and immune function [45].

Previous studies have investigated the association between manganese and type 2 diabetes using varying methods of assessing manganese status. Du et al. observed an inverse relationship between manganese intake and type 2 diabetes (independent of total antioxidant capacity) in two prospective cohort studies of Chinese individuals [25]. Similar results were observed by Mancini et al. and Gong et al., who investigated manganese intake and risk of type 2 diabetes in all women and postmenopausal women, respectively [23,46]. Eshak et al. examined these associations in a Japanese cohort, observing only a significant inverse association between manganese intake and risk of type 2 diabetes in women (but not men) [24]. The sex difference in these observed results was attributed to women’s higher absorption, bioavailability, and retention of manganese. Women typically have lower iron intake and an increased risk of low ferritin levels and iron deficiency; therefore, manganese does not have to compete with iron for absorption [24]. Other studies have examined relationships between manganese and type 2 diabetes using blood, urine, and serum manganese. Koh et al. observed that low blood manganese levels were associated with increased prevalence of type 2 diabetes in a cross-sectional study in a Korean population [47]. Yang et al. investigated associations between both blood and urinary manganese levels and markers of glucose metabolism and insulin traits [48]. Results showed a positive linear relationship between urinary manganese (but not blood manganese) with FPG and HbA1c among women, while a J-shaped nonlinear relationship of blood manganese with HOMA-IR and insulin among men [48]. Interestingly, Shan et al. observed a U-shaped association between serum manganese and type 2 diabetes in a Chinese population, suggesting that both low and high levels of manganese increase the risk of type 2 diabetes [49]. Evidence suggests that there is likely a link between decreased habitual manganese intake and increased risk of type 2 diabetes, which appears to be stronger in women and Asian populations [24,25,47,48]. The present study was the first to investigate the associations of dietary manganese intake and glucose metabolism/insulin traits in the unique cohort of individuals after an attack of AP. We found that manganese intake had an inverse relationship with both HbA1c and FPG in those with NODAP. Specifically, every 1 mg decrease in manganese intake was significantly associated with a 0.17 mmol/mol increase in HbA1c and a 0.02 mmol/L increase in FPG in people with NODAP. By studying the associations of both HbA1c and FPG, we were able to investigate the relationship between manganese intake and glucose metabolism comprehensively. HbA1c measures blood glucose levels over the past 90–120 days and therefore mitigates any day-to-day variation in plasma glucose levels. However, HbA1c can be affected by abnormal haemoglobin levels [50]. FPG is specific to plasma glucose after a fasted period (8 h in the present study) and remains unaffected by these abnormalities [51].

The mechanistic link between manganese and HbA1c and FPG is not fully understood; however, there is a possible role of the involvement of superoxide dismutase (SOD) enzymes [45,52,53]. There are three forms of SOD in mammals and manganese is a crucial component of manganese SOD (MnSOD) (it is worth noting that two of the other studied minerals—copper and zinc—are structural components of copper/zinc and extracellular SOD) [54]. SODs contribute to metabolic processes and protect cells against oxidative damage [45,52]. It has been hypothesised that MnSOD can affect glucose metabolism and insulin secretion [45]. MnSOD acts as an antioxidant to reduce oxidative stress and free radicals by catalysing the disproportionate superoxide anion radicals to hydrogen peroxide and molecular oxygen [45,52,53]. Reactive oxidant species and oxidative stress can result in impaired islet β-cell function, cause insulin resistance, and finally lead to impaired glucose metabolism [45]. Animal models have observed that manganese supplementation can increase MnSOD activity and improve glucose tolerance [55,56]. There are few studies on these associations in humans. Hope et al. observed that moderate to high intake of black tea (which is high in manganese) did not significantly alter circulating manganese levels or expression of leucocyte MnSOD [57]. However, an inverse relationship was noted between blood manganese and leucocyte MnSOD expression, which suggests that low levels of manganese may lead to overcompensation of MnSOD expression [57]. AP is a disease characterised by acute inflammation and oxidative stress, with subclinical low-grade inflammation persisting after the initial attack [58,59]. This leads to elevated oxidant levels and, consequently, MnSOD may be upregulated to manage oxidative damage [60]. Ściskalska et al. observed that patients with AP had a 3-fold increased MnSOD in erythrocytes compared with healthy controls and decreased plasma MnSOD, suggesting migration of MnSOD from other cells circulating in plasma (e.g., leukocytes and platelets) in the state of oxidative stress induced by AP [54]. Gut hormones (e.g., gastric inhibitory peptide and peptide YY) appear to increase circulating levels of pro-inflammatory cytokines, leading to persistent subclinical inflammation following an attack of AP [58,59]. As inverse associations between manganese intake and HbA1c and FPG were observed in the present study, there may be a link between manganese intake and MnSOD levels in patients after AP, perpetuating glucose metabolism dysfunction. Purposely designed studies are now warranted to investigate the exact mechanism behind the association between manganese intake and NODAP. In the present study, the mean manganese intakes were 2.91 and 2.46 mg/day for men and women, respectively. These mean manganese intakes are 47.1% and 50.8% lower than the New Zealand and Australia adequate intake guidelines of 5.5 and 5 mg/day (for men and women, respectively) [43]. Therefore, manganese intake meeting the adequate intake may be beneficial for people after an attack of AP. Manganese is present in a wide range of foods and food groups, including shellfish (1.1–6.8 mg/100 g), nuts (3.8–13 mg/100 g), whole grains (3.1–7 mg/100 g), legumes (0.40–2.5 mg/100 g), vegetables (0.7–1.5 mg/100 g), and black tea (0.4–1.9 mg/100 g) [61,62].

### 4.2. Iron Intake and Glucose Metabolism

Iron is a mineral that is an essential component of proteins (e.g., haemoglobin, myoglobin, and cytochromes) and a cofactor to enzymes involved in redox reactions [43]. Dietary iron has two forms (haem and non-haem) that differ in chemical structure, food sources, and absorptive properties. Non-haem iron, primarily derived from plant sources, is less bioavailable than haem iron (derived from meat products) as it is not as readily absorbed in the small intestine [63]. Iron absorption occurs via the apical brush border membrane of the small intestine by haem carrier protein (HCP1) and divalent metal transporter (DMT1), which enable transmembrane transport of haem iron into enterocytes, where iron is transported into plasma via ferroportin [64,65]. These transporters allow haem iron to be efficiently absorbed in the small intestine; however, non-haem iron forms insoluble non-absorbable complexes in an alkaline environment, thus requiring ferric iron to be reduced to ferrous iron to be absorbed [65,66]. The bioavailability of non-haem iron can also be limited by the presence of oxalates, phytates, polyphenols, phosphates, and calcium, which interfere with iron absorption. These compounds are present in most non-meat sources of iron; therefore, they primarily implicate non-haem iron absorption [65]. Iron homeostasis is tightly regulated. A peptide hormone, hepcidin, is the primary regulator of iron homeostasis by maintaining the systemic balance of iron storage, distribution, and utilisation [66]. Hepcidin negatively controls iron efflux by inactivating ferroportin in macrophages, enterocytes, and other cells to decrease plasma iron levels [64]. Hepcidin is upregulated in response to high iron levels and is down-regulated during iron deficiency, anaemia, or hypoxia to increase iron uptake [67]. Inflammatory states also lead to upregulation of hepcidin, triggered by proinflammatory cytokines such as interleukin-6 [64].

There is evidence to suggest a relationship between increased iron intake and impaired glucose metabolism resulting in an increased risk of type 2 diabetes [26,27,28,29,30,31], gestational diabetes [68,69,70], and metabolic syndrome [71,72]. Increased frequency of diabetes has also been observed in iron overload disorders (haemochromatosis and β-thalassemia), attributed to insulin resistance and destruction of pancreatic β-cells [64,73,74]. However, there is limited research on the effects of iron on glucose metabolism in individuals after pancreatitis. A previous ANDROMEDA study by the COSMOS group investigated associations between dietary iron and markers of glucose metabolism in individuals after pancreatitis. Kimita et al. found that total and non-haem iron were significantly inversely associated with FPG in individuals following AP [75]. These results contrast previous findings from studies investigating non-haem iron intake and glucose metabolism that found positive associations or no associations [71,72,76]. The present study found total iron intake in the NODAP group was significantly less than in the NAP group and was inversely associated with insulin sensitivity (HOMA-S). Every 1mg increase in total iron intake was significantly associated with a 3.12% decrease in HOMA-S in people with NODAP. These findings provide new insight into the role of iron intake on insulin sensitivity in people with NODAP. As our previous study focused on iron metabolism and hyperglycaemia in all individuals following AP, the subgroup analysis by diabetes type in the present study uncovered novel insights into iron’s role in NODAP.

However, the mechanism of this association is not fully understood [77]. In the context of type 2 diabetes, elevated levels of serum ferritin were associated with an increased risk of diabetes and were significantly associated with elevated levels of insulin, glucose, and HbA1c [78,79,80]. It has also been suggested that elevated ferritin levels in type 2 diabetes are due to inflammatory mechanisms rather than iron overload as there were no differences in transferrin receptor levels [81,82]. A comprehensive review of iron metabolism and the exocrine pancreas provided evidence for crosstalk between iron metabolism and the exocrine pancreas [83]. Chand et al. found that hepcidin levels were significantly increased and ferritin levels significantly decreased in participants with prediabetes/diabetes after AP, providing further evidence that iron may be involved in and the pathogenesis of NODAP [77]. In the present study, the mean iron intake for men was 10.51 mg/day, which is 75% higher than the New Zealand and Australia EAR of 6 mg/day for men [43]. The mean intake of women in our study cohort was 8.87 mg/day, which is 77.4% higher than the New Zealand and Australia EAR of 5 mg/day for women over 51 [43]. Dietary sources of haem iron include seafood (1.1–16.9 mg/100 g) and meat (2.3–21 mg/100 g), while sources of non-haem iron include fortified grain products (2.6–15.4 mg/100 g), legumes (5.3–6.4 g/100 g), nuts (3.5–9.2 mg/100 g), and vegetables (0.6–4.4 mg/100 g) [62]. In New Zealand, whole grain cereals, meat, fish, and poultry are significant contributors to iron intake [43,84]. Therefore, iron intake within the recommended range may be beneficial for people after an attack of AP.

### 4.3. Iodine and Selenium Intakes and Glucose Metabolism

The pairing of selenium and iodine and their involvement in glucose metabolism has been suggested [85]. Iodine plays an important role in the synthesis of thyroid hormones—triiodothyronine (T3) and thyroxine (T4)—and is therefore crucial for the regulation of basal metabolic rate, macronutrient metabolism, redox reactions, and normal growth and development within the body [86,87]. Iodine is ingested in different chemical forms, which are absorbed differently. Up to 90% of iodide is absorbed in the stomach and duodenum, while iodate is reduced to iodide for absorption. The rate of iodine absorption is dependent on the iodine status of an individual. In individuals with adequate iodine levels, up to 10% of absorbed iodine is taken up by the thyroid, whereas as much as 80% can be taken up in iodine deficiency (62). In the thyroid, thyroperoxidase and hydrogen peroxide oxidise iodide bind with thyroglobulin to produce thyroid hormone precursors, monoiodotyrosine and diiodotyrosine, which then form T3 and T4 [87]. Iodine not taken up by the thyroid is excreted from the body in urine [88]. Due to its role in thyroid hormone synthesis, dietary iodine intake is closely related to thyroid function [89,90]. Both iodine deficiency and iodine excess have been associated with an increased risk of thyroid disorders including iodide-induced hyperthyroidism, autoimmune thyroid disease, iodine-induced hypothyroidism [90,91].

There is a clear link between thyroid function, diabetes and glucose metabolism due to thyroid hormones’ role in regulating carbohydrate metabolism and pancreatic function [92,93]. Studies have found both hypo- and hyperthyroidism have been associated with insulin resistance and impaired glycaemic control, leading to hyperglycaemia [92,93,94,95]. Therefore, a link between iodine intake and type 2 diabetes is plausible. However, few studies focused on associations between dietary iodine intake and type 2 diabetes. Mancini et al. found iodine intake above 160 μg/day was significantly associated with an increased risk of type 2 diabetes in French women [96]. Other studies have investigated the same association using urinary iodine—a commonly used indicator of iodine intake [88]. Liu et al. examined the effect of excessive iodine intake on blood glucose levels in Chinese adults [86]. The cross-sectional study assessed median water iodine concentration (MWIC) and median urinary iodine concentration (MUIC) in in three geographical areas classed as iodine-adequate (MWIC 6.3 μg/L MUIC 126.6 μg/L), iodine-sufficient (MWIC 79.8 μg/L, MUIC 221.2 μg/L), and iodine-excess (MWIC 506.0 μg/L, MUIC 421.3 μg/L) [86]. The authors found that blood glucose of adults in iodine-sufficient and iodine-excess areas was increased, compared with the iodine-adequate area. Urinary iodine, thyroid stimulating hormone (TSH), and free T4 also had a nonlinear correlation with blood glucose [86]. Therefore, it was concluded that excessive iodine intake might result in elevated blood glucose and contribute to the development of diabetes. The present study found iodine intake is significantly associated with HbA1c levels in people with NODAP. Every 1 μg increase in iodine intake was significantly associated with a 0.17 mmol/mol increase in HbA1c in those with NODAP.

Selenium plays structural and enzymatic roles in antioxidant defence systems throughout the body [33,97,98]. It is a component of selenoproteins that act as a cofactor of many enzymes, including glutathione peroxides, thyroid peroxidases, thioredoxin reductases, and iodothyronine deiodinidases [33,97]. Selenium’s role in diabetes has been debated. It was previously hypothesised that selenium might prevent the development of diabetes due to its antioxidant properties [99]. Selenate has also been observed to act as an effective insulin-mimetic by stimulating glycolysis, fatty acid synthesis and, in some cases, glycogen synthesis in animal models [100,101]. However, in human studies, there is evidence to suggest that increased selenium intake has a positive relationship with the risk of type 2 diabetes. Siddiqi et al. observed a significant direct association between dietary selenium intake and HbA1c and FBG [33]. These results were consistent with Wei et al., who found a significant positive association between dietary selenium and the prevalence of diabetes in a Chinese population [34]. A prospective study involving 7182 Italian women also found that dietary selenium had a strong association with type 2 diabetes [32]. Selenium supplementation has also been studied in the context of diabetes. Stranges et al. examined the effect of long-term selenium supplementation and diabetes in low-selenium regions of eastern United States [102]. Results showed that increased selenium intake (via 200 μg supplementation) significantly increased the cumulative incidence of T2DM in this population compared with the placebo group [102]. Therefore, evidence suggests limited usefulness of selenium supplementation in the prevention of T2DM. One study showed a U-shape association between dietary selenium intake and type 2 diabetes, which indicates that low intake may also increase the risk of T2DM [103]. Behar et al. observed that high and low dietary selenium intake could impact glycaemic control in women from Algeria [103]. In that population, both the lowest and highest quintiles of selenium intake were associated with a significant increase in HbA1c [103]. However, a systematic review and meta-analysis of selenium exposure (measured in serum, plasma, whole blood, nail, urine, hair, tears, and dietary intake) and risk of diabetes found a consistent pattern of a positive association in both nonexperimental and experimental studies, with limited evidence for associations between low selenium exposure and diabetes [104]. Our study found an inverse relationship between habitual selenium intake and insulin sensitivity (but not HbA1c or FPG) in people with NODAP. Every 1 μg increase in selenium intake was significantly associated with a 1.71% decrease in HOMA-S (%).

Overall, our results suggest that selenium and iodine intake play a role in insulin sensitivity, which may be involved with the progression of NODAP. A possible mechanism for this is their respective roles in the synthesis of thyroid hormones. Thyroid hormones are efficient modulators of catabolism of energy sources (including carbohydrates) and both hyperthyroidism and hypothyroidism have been associated with the development of diabetes [105]. A positive relationship has been observed between thyroid stimulating hormone and HOMA-IR, indicating hypothyroidism has a role in insulin resistance and diabetes [106,107]. In AP, serum free T3 and T4 are reduced and TSH levels are increased. It has also been observed that levels of TSH are related to severity of AP [108,109,110]. Therefore, it is possible that iodine and selenium requirements may be altered for people with a history of AP. Dietary intake of both selenium and iodine varies greatly and is often dependent on geographical location and soil composition. Mean iodine intake in the present study was 119.61 µg/day in men and 104.93 µg/day in women, which is 20% and 5% higher than the New Zealand and Australia EAR or 100 µg/day for men and women [43]. The native iodine content of most foods and beverages is low due to the lack of iodine in New Zealand soil. Therefore, the majority of iodine intake is from fortified foods (commercially made bread 30.1–53.5 µg/100 g and iodised salt 32–64 µg/100 g) [43,111]. Seafood (12–370 µg/100 g), eggs (61 µg/100 g), and milk products (10–50.4 µg/100 g) are also sources of iodine [43,62,112]. The iodine content of meat products (4.2–50 µg/100 g) is reflective of the iodine content of animal feed used [62,87]. The use of processing aids (e.g., calcium iodate, potassium iodate, potassium iodide, and cuprous iodide) also increases iodine content in processed foods [43,87]. In our study population, mean selenium intake was 59.73 µg/day in men and 49.97 µg/day in women—in line with the New Zealand and Australia EAR of 60 µg/day and 50 µg/day for men and women [43]. Dietary sources of selenium include Brazil nuts (1270 µg/100g), seafood (46.7–142 µg/100 g), meat (21–110 µg/100 g), whole grains (7.6–28.7 µg/100 g), and vegetables (0.9–16.1 µg/100 g) [62]. However, plant sources of selenium are not as efficient as animal products due to their high water content and the varying soil content of selenium [43,97]. Dietary factors such as vitamins, fat, protein, and some heavy metals also alter the bioavailability of selenium [113]. Therefore, the requirements of these minerals for people after AP may be different than for the general population.

### 4.4. Limitations

Findings of the present study must be considered with several limitations. First, habitual dietary intake of minerals was ascertained using a self-reported FFQ, which relies on the ability of respondents to recall their usual intake of foods. Therefore, FFQ data might be biased due to omission or addition of foods, and over- or under-estimating frequency and portion of foods [114]. However, the EPIC-Norfolk FFQ has been extensively validated and it provides a more accurate estimation of long-term habitual intake of minerals compared with other dietary assessment methods (e.g., 3-day food records) [38,115]. Second, the possibility of changes in the habitual intake after an attack of AP must not be discounted. However, the FFQ assesses habitual intake in the 12 months before the study visit and our study participants were recruited, on average, in 26 months since the last attack of AP (hence, the data captured in the FFQ focused on the period after the AP attack). Also, they were not encouraged to make any dietary changes after hospital discharge. Third, the present study investigated intake of each mineral in isolation and did not account for all dietary covariates associated, including but not limited to intake of other minerals, protein, fat, and carbohydrates. Due to the complex composition of food, individuals consume various macronutrients and micronutrients at one time, some of which may interact with absorption or utilisation of another. For example, iron absorption is known to be influenced by many factors that inhibit (calcium, zinc, manganese, phytates, polyphenols, and vegetable protein) or enhance (meat, fish, poultry factor, vitamin C and citric, lactic, and malic acids) it [63]. Various minerals are also known to compete for absorption. For example, manganese bioavailability is influenced by dietary iron intake, as they compete for binding to transferrin in serum and transport by divalent metal transporter 1 [116]. Calcium, phosphate, and zinc are also known to interact with manganese absorption [43,44,117]. Considering the relatively small sample size of the present study, including these covariates might have resulted in the overfitting of statistical models. However, energy intake was included in statistical models to encompass most dietary variables as a single factor, along with other covariates (age, sex, V/S fat volume ratio, smoking status, alcohol intake, aetiology of AP, number of AP episodes, cholecystectomy, use of antidiabetic medications) to provide robust models. It is also worth noting that V/S fat volume ratio was used instead of traditional measures of adiposity (BMI and waist circumference) as it is a more comprehensive measure of relative body fat distribution and is correlated superbly to metabolic risk [118]. Fourth, use of oral pharmacologic agents and/or insulin therapy was exclusive to those in the T2DM group and not in the NODAP group. Therefore, use of these medications has the potential to confound results, particularly between markers of glucose metabolism and mineral intake in the T2DM group. By adjusting for antidiabetic medication use in statistical models, internal validity of results is maintained and results are comparable between study groups. Future research should further investigate the impact of antidiabetic medications on mineral intake and markers of glucose metabolism. This study also did not assess other possible confounders (such as enzyme activity, levels of hormones or inflammatory markers), which may have implications on glucose metabolism and insulin traits following AP. They should be addressed in further research. Fifth, dietary supplements were not included in this study due to limitations of the FETA software [38]. Hence, results in the present study can only be applied to habitual intake of minerals, which may be altered by supplement use. Future studies should investigate the associations of supplements on people with NODAP. Sixth, our study only investigated dietary intake of minerals, which is not reflective of mineral status. Therefore, we cannot assess whether participants nutritional status is sufficient, deficient, or excessive. As mineral status of an individual may affect glucose metabolism and insulin traits, future research should use appropriate methods of assessment (plasma, whole blood, urine, nail and/or hair tests). Last, due to the cross-sectional design of this study, a causal relationship between dietary mineral intake and markers of glucose metabolism and insulin traits cannot be inferred. However, this was the first study investigating this relationship in individuals with NODAP. Insights from the present study will help design future prospective longitudinal studies of dietary mineral intake in people after an attack of pancreatitis.

## 5. Conclusions

Of the 13 minerals investigated in the present study, intake of iron, nitrogen, phosphorous, and zinc was significantly altered in people with NODAP. These people were also characterised by significant inverse associations between intake of manganese and both HbA1c and FPG, as well as intake of iron and HOMA-S. Iodine intake was significantly directly associated with HbA1c levels whereas intake of selenium was significantly inversely associated with HOMA-S. These findings give light to possible role of mineral intake in NODAP. Longitudinal studies and randomised controlled trials are now warranted to investigate possible causal relationships and mechanisms of mineral intake on NODAP to provide evidence for nutritional interventions specifically for people at risk of NODAP.

## Figures and Tables

**Table 1:** Characteristics of the study cohort.

Characteristic	Total	NODAP	T2DM	NAP	*p* *
	(*n* = 106)	(*n* = 37)	(*n* = 37)	(*n* = 32)	
Age	56.1 (14.5)	58.9 (14.4)	57.2(15.0)	51.6(13.3)	0.094
Sex					** 0.031 **
Men	69 (65.1)	26 (70.3)	28 (75.7)	15 (46.9)	
Women	37 (34.9)	11 (29.7)	9 (24.3)	17 (53.1)	
Daily energy intake (kcal)	1686 (609)	1776 (692)	1728 (534)	1534 (574)	0.226
V/S fat volume ratio	0.77 (0.43)	0.81 (0.40)	0.87 (0.46)	0.61 (0.40)	** 0.035 **
Alcohol intake (g/day)	11.1 (17.9)	13.4 (21.9)	8.7 (13.1)	11.1 (17.7)	0.527
Smoking status					0.052
Never	47 (44)	11 30)	21 (57)	15 (47)	
Former	35 (33)	16 (4)	11 (30)	8 (25)	
Light (<20 ^a^/d)	8 (8)	3 (8)	2 (5)	3 (9)	
Moderate (20–39 ^a^/d)	15 (14)	7 (19)	2 (5)	6 (19)	
Heavy (>39 ^a^/d)	0 (0)	0 (0)	0 (0)	0 (0)	
Aetiology of AP					0.563
Biliary	40 (38)	14 (38)	14 (38)	12 (38)	
Alcohol-related	21 (20)	12 (32)	5 (14)	4 (13)	
Other	45 (43)	11 (30)	18 (49)	16 (50)	
Number of AP episodes	1.9 (2.8)	2.3 (3.8)	1.4 (1.0)	1.8 (2.8)	0.434
Cholecystectomy					0.538
No	66 (62)	24 (65)	25 (68)	17 (53)	
Yes	39 (37)	13 (35)	12 (32)	14 (44)	
Use of anti-diabetic medications					** <0.001 **
None	92 (87)	37 (100)	23 (62)	32 (100)	
Oral medication	8 (8)	0 (0)	8 (22)	0 (0)	
Insulin	6 (6)	0 (0)	6 (16)	0 (0)	
HbA1c (mmol/mol)	40.61 (10.82)	39.05 (4.80)	47.19 (15.23)	34.61 (2.55)	** <0.001 **
Fasting plasma glucose (mmol/L)	5.86 (1.74)	5.86 (0.92)	6.61 (2.55)	4.96 (0.34)	** <0.001 **
Fasting insulin (mU/L)	16.68 (36.01)	12.98 (9.96)	24.62 (59.95)	12.15 (10.27)	0.277
HOMA-S (%)	0.88 (0.74)	1.02 (1.06)	0.72 (0.44)	0.90 (0.49)	0.228
HOMA-β (%)	106.97 (56.87)	95.74 (45.63)	103.24 (57.12)	125.07 (65.87)	0.098

Abbreviations: NODAP = New-onset diabetes or prediabetes after acute pancreatitis. T2DM = Type 2 diabetes or prediabetes prior to acute pancreatitis. NAP = Normoglycaemia after acute pancreatitis. AP = Acute pancreatitis. V/S fat volume ratio = Visceral to subcutaneous fat volume ratio. HbA1c = glycated haemoglobin. HOMA-β = homeostasis model assessment of β-cell dysfunction. HOMA-S homeostasis model assessment of insulin sensitivity. Data are presented as mean (standard deviation) or frequency(percentage). ^a^ cigarettes per day. * *p* values were calculated from one way ANOVA. Significance was set at *p* < 0.05. Significant values are shown in bold.

**Table 2:** Associations between habitual mineral intake and the study groups.

Mineral	Model	T2DM				NODAP			
		β	*p*	95% CI		β	*p*	95% CI	
				Lower	Upper			Lower	Upper
Calcium (mg)	1	0.009	0.821	−0.070	0.089	−0.023	0.565	−0.102	0.056
	2	−0.020	0.537	−0.082	0.043	−0.059	0.063	−0.120	0.003
	3	−0.018	0.581	−0.080	0.045	−0.058	0.064	−0.120	0.003
	4	−0.022	0.506	−0.089	0.044	−0.057	0.073	−0.120	0.005
	5	−0.010	0.797	−0.087	0.067	−0.056	0.091	−0.121	0.009
Chloride (mg)	1	0.047	0.326	−0.047	0.141	0.040	0.396	−0.053	0.133
	2	0.000	0.996	−0.057	0.057	−0.020	0.471	−0.077	0.036
	3	0.003	0.926	−0.054	0.060	−0.020	0.471	−0.077	0.036
	4	0.002	0.939	−0.057	0.061	−0.023	0.417	−0.079	0.033
	5	0.024	0.492	−0.045	0.093	−0.025	0.394	−0.084	0.033
Copper (mg)	1	0.123	**0.012**	0.027	0.219	0.090	0.064	−0.005	0.185
	2	0.073	**0.039**	0.004	0.141	0.026	0.445	−0.042	0.094
	3	0.078	**0.026**	0.010	0.146	0.026	0.436	−0.041	0.093
	4	0.072	**0.049**	0.000	0.144	0.026	0.458	−0.043	0.094
	5	0.061	0.147	−0.022	0.145	0.019	0.592	−0.052	0.090
Iodine (μg)	1	0.046	0.259	−0.034	0.126	0.017	0.675	−0.063	0.097
	2	0.030	0.402	−0.040	0.099	−0.007	0.846	−0.076	0.062
	3	0.037	0.269	−0.029	0.104	−0.007	0.843	−0.073	0.059
	4	0.015	0.666	−0.054	0.084	−0.009	0.787	−0.074	0.056
	5	0.010	0.811	−0.071	0.091	0.000	0.990	−0.069	0.068
Iron (mg)	1	0.012	0.785	−0.076	0.101	−0.002	0.963	−0.090	0.086
	2	−0.037	0.205	−0.096	0.021	−0.064	** 0.029 **	−0.122	−0.007
	3	−0.036	0.220	−0.095	0.022	−0.064	** 0.030 **	−0.122	−0.006
	4	−0.034	0.272	−0.094	0.027	−0.061	** 0.036 **	−0.119	−0.004
	5	−0.034	0.342	−0.104	0.036	−0.076	** 0.013 **	−0.135	−0.016
Magnesium (mg)	1	0.025	0.513	−0.051	0.100	0.034	0.371	−0.041	0.109
	2	−0.013	0.582	−0.062	0.035	−0.013	0.581	−0.061	0.035
	3	−0.011	0.665	−0.059	0.038	−0.013	0.579	−0.061	0.034
	4	−0.015	0.559	−0.066	0.036	−0.013	0.600	−0.061	0.035
	5	−0.016	0.589	−0.076	0.043	−0.017	0.516	−0.067	0.034
Manganese (mg)	1	0.022	0.687	−0.085	0.128	0.028	0.600	−0.078	0.134
	2	−0.006	0.878	−0.089	0.076	−0.017	0.687	−0.098	0.065
	3	−0.003	0.942	−0.085	0.079	−0.017	0.688	−0.098	0.065
	4	−0.021	0.633	−0.107	0.065	−0.020	0.634	−0.101	0.062
	5	−0.049	0.343	−0.150	0.053	−0.035	0.418	−0.121	0.051
Nitrogen (g)	1	0.010	0.784	−0.060	0.079	−0.025	0.474	−0.094	0.044
	2	−0.023	0.299	−0.066	0.021	−0.066	** 0.003 **	−0.109	−0.023
	3	−0.021	0.346	−0.064	0.023	−0.066	** 0.003 **	−0.109	−0.023
	4	−0.042	0.055	−0.086	0.001	−0.065	** 0.002 **	−0.106	−0.024
	5	−0.033	0.205	−0.084	0.018	−0.066	** 0.003 **	−0.110	−0.023
Phosphorous (mg)	1	0.017	0.610	−0.048	0.081	−0.005	0.880	−0.069	0.059
	2	−0.017	0.291	−0.050	0.015	−0.046	** 0.005 **	−0.078	−0.014
	3	−0.015	0.351	−0.047	0.017	−0.046	** 0.005 **	−0.078	−0.015
	4	−0.026	0.124	−0.059	0.007	−0.045	** 0.005 **	−0.077	−0.014
	5	−0.023	0.241	−0.061	0.015	−0.046	** 0.006 **	−0.078	−0.014
Potassium (mg)	1	0.017	0.642	−0.055	0.088	0.020	0.578	−0.051	0.091
	2	−0.019	0.449	−0.069	0.031	−0.025	0.318	−0.074	0.024
	3	−0.015	0.543	−0.064	0.034	−0.025	0.310	−0.073	0.023
	4	−0.018	0.492	−0.069	0.034	−0.024	0.324	−0.073	0.024
	5	−0.020	0.510	−0.080	0.040	−0.026	0.322	−0.076	0.025
Selenium (μg)	1	0.058	0.202	−0.032	0.147	0.035	0.439	−0.054	0.123
	2	0.024	0.445	−0.038	0.085	−0.011	0.716	−0.072	0.049
	3	−0.011	0.713	−0.071	0.049	−0.011	0.713	−0.071	0.049
	4	0.009	0.780	−0.053	0.071	−0.014	0.637	−0.073	0.045
	5	−0.008	0.828	−0.081	0.065	−0.011	0.722	−0.072	0.050
Sodium (mg)	1	0.042	0.380	−0.053	0.137	0.030	0.536	−0.065	0.124
	2	−0.004	0.895	−0.062	0.054	−0.031	0.281	−0.089	0.026
	3	−0.002	0.953	−0.060	0.056	−0.031	0.281	−0.088	0.026
	4	−0.005	0.875	−0.065	0.055	0.009	0.780	−0.053	0.071
	5	0.022	0.537	−0.048	0.092	−0.034	0.256	−0.094	0.025
Zinc (mg)	1	0.025	0.489	−0.046	0.096	−0.024	0.510	−0.094	0.047
	2	−0.017	0.459	−0.063	0.029	−0.071	** 0.002 **	−0.116	−0.026
	3	−0.017	0.467	−0.063	0.029	−0.071	** 0.003 **	−0.117	−0.026
	4	−0.029	0.232	−0.077	0.019	−0.070	** 0.003 **	−0.116	−0.025
	5	−0.017	0.527	−0.071	0.037	−0.078	** 0.001 **	−0.124	−0.033

Abbreviations: NODAP = New-onset diabetes or prediabetes after acute pancreatitis. T2DM = Type 2 diabetes or prediabetes prior to acute pancreatitis. NAP = Normoglycaemia after acute pancreatitis. 95% CI = 95% confidence interval. Footnotes: Data are presented as β coefficients, 95% CI and *p* values (from ANCOVA analysis). NAP group was used as the reference group. All the variables were log-transformed. Model 1: unadjusted model. Model 2: age, sex, daily energy intake. Model 3: age, sex, daily energy intake, V/S fat volume ratio. Model 4: age, sex, daily energy intake, V/S fat volume ratio, alcohol intake, smoking status Model 5: age, sex, daily energy intake, V/S fat volume ratio, alcohol intake, smoking status, aetiology of AP, number of AP episodes, cholecystectomy, use of antidiabetic medications. Significance was set at *p* < 0.05. Significant values are shown in bold.

**Table 3:** Associations between habitual mineral intake and HbA1c in the study groups.

Mineral	Model	NAP					T2DM					NODAP				
		R^2^	Unstandardised	*p*	95% CI		R^2^	Unstandardised	*p*	95% CI		R^2^	Unstandardised	*p*	95% CI	
			B		Lower	Upper		B		Lower	Upper		B		Lower	Upper
Calcium (mg)	1	0.003	0.695	0.772	−4.164	5.554	0.028	−15.434	0.368	−49.861	18.994	0.055	8.423	0.164	−3.610	20.456
	2	0.278	0.404	0.886	−5.315	6.123	0.039	−16.023	0.528	−67.269	35.222	0.170	12.012	0.172	−5.491	29.515
	3	0.280	0.452	0.875	−5.397	6.301	0.081	−15.410	0.541	−66.347	35.528	0.190	14.180	0.124	−4.111	32.470
	4	0.347	1.608	0.595	−4.558	7.774	0.173	−22.037	0.380	−72.650	28.576	0.247	13.916	0.131	−4.383	32.216
	5	0.382	1.344	0.683	−5.415	8.103	0.493	−14.339	0.568	−65.521	36.844	0.275	12.586	0.199	−7.032	32.204
Chloride (mg)	1	0.085	4.268	0.112	−1.059	9.596	0.044	−15.515	0.227	−41.158	10.127	0.051	5.530	0.179	−2.658	13.718
	2	0.323	5.032	0.198	−2.798	12.863	0.063	−24.427	0.325	−74.324	25.471	0.126	4.013	0.603	−11.565	19.590
	3	0.341	6.349	0.139	−2.201	14.900	0.109	−24.191	0.326	−73.745	25.364	0.133	4.323	0.582	−11.515	20.160
	4	0.382	5.548	0.217	−3.484	14.580	0.222	−32.960	0.184	−82.570	16.650	0.191	3.785	0.633	−12.267	19.837
	5	0.424	5.932	0.215	−3.720	15.585	0.484	−11.699	0.619	−59.769	36.371	0.233	3.655	0.657	−13.081	20.391
Copper (mg)	1	0.042	2.683	0.268	−2.177	7.544	0.022	−13.209	0.390	−44.091	17.672	0.004	1.427	0.706	−6.181	9.035
	2	0.298	3.412	0.386	−4.547	11.371	0.037	−8.386	0.677	−49.121	32.349	0.151	−6.212	0.283	−17.795	5.370
	3	0.303	3.655	0.368	−4.553	11.863	0.079	−1.616	0.938	−43.979	40.746	0.153	−5.948	0.317	−17.864	5.968
	4	0.343	1.698	0.701	−7.325	10.722	0.170	−3.533	0.864	−45.511	38.445	0.208	−5.829	0.356	−18.551	6.893
	5	0.379	1.663	0.785	−10.893	14.218	0.478	0.148	0.994	−37.444	37.740	0.242	−4.840	0.472	−18.487	8.807
Iodine (μg)	1	0.050	3.050	0.226	−1.998	8.098	0.040	−21.520	0.247	−58.669	15.629	0.065	7.667	0.129	−2.340	17.674
	2	0.298	2.446	0.394	−3.351	8.244	0.052	−18.384	0.425	−64.815	28.047	0.185	9.758	0.116	−2.534	22.049
	3	0.301	2.571	0.382	−3.382	8.524	0.084	−10.465	0.667	−59.635	38.706	0.241	14.951	** 0.037 **	0.997	28.905
	4	0.359	2.552	0.401	−3.613	8.717	0.181	−14.979	0.533	−63.706	33.747	0.276	14.135	0.065	−0.929	29.200
	5	0.390	2.185	0.520	−4.771	9.140	0.498	−19.879	0.350	−62.977	23.219	0.354	17.763	** 0.032 **	1.601	33.926
Iron (mg)	1	0.017	1.807	0.490	−3.479	7.093	0.065	−22.774	0.140	−53.395	7.848	0.004	1.644	0.697	−6.852	10.139
	2	0.278	−0.183	0.959	−7.449	7.082	0.099	−41.281	0.146	−97.747	15.184	0.153	−7.510	0.264	−20.979	5.958
	3	0.279	−0.041	0.991	−7.555	7.473	0.139	−39.418	0.163	−95.710	16.874	0.159	−7.557	0.268	−21.211	6.098
	4	0.348	2.304	0.562	−5.805	10.412	0.202	−30.213	0.294	−88.098	27.672	0.205	−6.118	0.392	−20.529	8.294
	5	0.392	3.614	0.477	−6.799	14.027	0.478	−3.564	0.893	−57.916	50.788	0.242	−5.279	0.478	−20.352	9.794
Magnesium (mg)	1	0.015	2.080	0.517	−4.410	8.570	0.029	−16.583	0.329	−50.606	17.439	0.000	−0.035	0.995	−10.529	10.459
	2	0.278	0.748	0.872	−8.730	10.226	0.045	−17.348	0.523	−72.103	37.408	0.198	−15.774	0.084	−33.813	2.264
	3	0.281	1.328	0.794	−9.013	11.668	0.090	−16.465	0.541	−70.912	37.982	0.199	−15.595	0.101	−34.382	3.193
	4	0.342	1.603	0.752	−8.779	11.985	0.182	−17.570	0.509	−71.398	36.259	0.241	−14.792	0.152	−35.363	5.779
	5	0.377	0.706	0.900	−10.879	12.292	0.481	−9.455	0.698	−59.159	40.248	0.291	−15.995	0.138	−37.481	5.492
Manganese (mg)	1	0.035	2.419	0.311	−2.381	7.220	0.003	3.558	0.751	−19.041	26.157	0.041	−4.493	0.230	−11.951	2.965
	2	0.322	4.217	0.201	−2.395	10.829	0.070	16.181	0.274	−13.505	45.866	0.338	−15.097	**0.003**	−24.555	−5.638
	3	0.332	4.740	0.172	−2.197	11.678	0.117	16.108	0.273	−13.362	45.579	0.338	−15.274	**0.003**	−25.116	−5.431
	4	0.365	3.708	0.341	−4.186	11.602	0.183	10.427	0.495	−20.504	41.359	0.411	−16.465	**0.002**	−26.546	−6.385
	5	0.399	4.551	0.392	−6.300	15.401	0.490	10.357	0.469	−18.745	39.458	0.455	−17.147	**0.003**	−27.829	−6.464
Nitrogen (g)	1	0.001	0.747	0.837	−6.634	8.128	0.030	−19.306	0.321	−58.323	19.711	0.017	3.968	0.440	−6.356	14.293
	2	0.297	−4.643	0.407	−15.959	6.673	0.048	−24.332	0.472	−92.587	43.924	0.119	0.040	0.996	−17.873	17.952
	3	0.297	−4.622	0.438	−16.695	7.450	0.092	−21.690	0.520	−89.766	46.385	0.125	0.611	0.946	−17.734	18.956
	4	0.366	−5.911	0.327	−18.122	6.301	0.169	−6.075	0.872	−82.534	70.384	0.184	−0.280	0.977	−19.817	19.257
	5	0.402	−6.015	0.366	−19.565	7.535	0.487	21.821	0.536	−50.047	93.689	0.227	0.707	0.944	−19.774	21.187
Phosphorous (mg)	1	0.010	1.936	0.593	−5.384	9.255	0.029	−20.931	0.329	−63.886	22.025	0.028	5.931	0.322	−6.066	17.928
	2	0.280	−1.931	0.777	−15.783	11.921	0.047	−28.990	0.485	−112.750	54.770	0.125	7.100	0.627	−22.399	36.598
	3	0.281	−1.597	0.824	−16.252	13.058	0.090	−24.752	0.550	−108.475	58.970	0.136	9.891	0.523	−21.318	41.100
	4	0.341	−2.105	0.776	−17.217	13.006	0.178	−23.107	0.582	−108.193	61.980	0.205	14.626	0.389	−19.608	48.860
	5	0.381	−2.991	0.706	−19.313	13.331	0.478	−5.990	0.885	−90.846	78.867	0.244	13.957	0.448	−23.277	51.190
Potassium (mg)	1	0.007	1.337	0.662	−4.851	7.526	0.036	−19.541	0.274	−55.273	16.190	0.001	1.188	0.845	−11.066	13.442
	2	0.278	0.318	0.935	−7.579	8.216	0.054	−24.484	0.404	−83.561	34.593	0.172	−14.573	0.160	−35.223	6.077
	3	0.280	0.700	0.867	−7.806	9.207	0.093	−19.581	0.507	−79.212	40.049	0.172	−14.359	0.190	−36.204	7.486
	4	0.342	1.503	0.721	−7.086	10.093	0.187	−22.375	0.442	−81.154	36.405	0.212	−11.737	0.321	−35.497	12.022
	5	0.378	0.938	0.838	−8.489	10.366	0.495	−22.591	0.387	−75.593	30.410	0.261	−13.299	0.286	−38.402	11.804
Selenium (μg)	1	0.040	2.931	0.282	−2.541	8.404	0.001	−3.726	0.836	−40.123	32.672	0.028	3.654	0.321	−3.718	11.025
	2	0.283	1.851	0.674	−7.101	10.803	0.042	15.057	0.575	−39.189	69.303	0.123	2.239	0.697	−9.372	13.850
	3	0.285	1.978	0.661	−7.198	11.155	0.117	31.274	0.272	−25.844	88.391	0.130	2.706	0.646	−9.201	14.612
	4	0.343	1.688	0.710	−7.586	10.963	0.215	39.157	0.218	−24.592	102.907	0.186	1.519	0.799	−10.596	13.633
	5	0.379	1.311	0.784	−8.516	11.139	0.487	17.826	0.540	−41.505	77.158	0.237	3.642	0.569	−9.322	16.607
Sodium (mg)	1	0.088	4.275	0.104	−0.940	9.490	0.040	−14.905	0.248	−40.714	10.904	0.052	5.413	0.175	−2.519	13.345
	2	0.334	5.458	0.150	−2.114	13.030	0.056	−21.295	0.389	−71.052	28.463	0.128	4.165	0.575	−10.800	19.130
	3	0.352	6.607	0.107	−1.542	14.757	0.102	−21.094	0.390	−70.514	28.327	0.134	4.312	0.567	−10.871	19.495
	4	0.390	5.738	0.179	−2.828	14.305	0.216	−31.273	0.214	−81.738	19.192	0.191	3.797	0.619	−11.648	19.243
	5	0.438	6.639	0.153	−2.688	15.966	0.480	−7.151	0.770	−57.207	42.906	0.235	4.181	0.597	−11.894	20.256
Zinc (mg)	1	0.000	0.059	0.986	−6.747	6.865	0.033	−19.770	0.295	−57.549	18.010	0.026	4.984	0.343	−5.549	15.517
	2	0.327	−6.886	0.179	−17.132	3.360	0.062	−29.825	0.329	−91.235	31.585	0.120	2.044	0.817	−15.812	19.899
	3	0.327	−7.089	0.193	−18.004	3.826	0.124	−36.619	0.231	−97.872	24.634	0.126	2.199	0.806	−15.918	20.315
	4	0.401	−8.146	0.134	−19.009	2.716	0.188	−25.215	0.431	−89.980	39.550	0.185	1.313	0.886	−17.316	19.943
	5	0.464	−12.169	0.085	−26.193	1.856	0.480	8.802	0.779	−55.273	72.876	0.227	0.970	0.919	−18.434	20.375

Abbreviations: NODAP = New-onset diabetes or prediabetes after acute pancreatitis. T2DM = Type 2 diabetes or prediabetes prior to acute pancreatitis. NAP = Normoglycaemia after acute pancreatitis. HbA1c = glycated haemoglobin. 95% CI = 95% confidence interval. Footnotes: Data are presented as R-squared values (from crude analysis), unstandardised B, *p* values (from linear regression) and 95% confidence intervals. All the variables were log-transformed. Model 1: unadjusted model. Model 2: age, sex, daily energy intake. Model 3: age, sex, daily energy intake, V/S fat volume ratio. Model 3: age, sex, daily energy intake, V/S fat volume ratio. Model 4: age, sex, daily energy intake, V/S fat volume ratio, alcohol intake, smoking status. Model 5: age, sex, daily energy intake, V/S fat volume ratio, alcohol intake, smoking status, aetiology of AP, number of AP episodes cholecystectomy, use of antidiabetic medications. Significance was set at *p* < 0.05. Significant values are shown in bold.

**Table 4:** Associations between habitual mineral intake and fasting plasma glucose in the study groups.

Mineral	Model	NAP					T2DM					NODAP				
		R^2^	Unstandardised	*p*	95% CI		R^2^	Unstandardised	*p*	95% CI		R^2^	Unstandardised	*p*	95% CI	
			B		Lower	Upper		B		Lower	Upper		B		Lower	Upper
Calcium (mg)	1	0.004	0.097	0.753	−0.527	0.720	0.021	−2.219	0.404	−7.561	3.124	0.090	2.072	0.071	−0.191	4.334
	2	0.151	0.149	0.703	−0.648	0.947	0.049	−2.695	0.492	−10.614	5.225	0.228	1.495	0.354	−1.740	4.730
	3	0.222	0.185	0.630	−0.598	0.967	0.088	−2.608	0.505	−10.511	5.296	0.234	1.722	0.311	−1.688	5.131
	4	0.225	0.192	0.651	−0.675	1.058	0.283	−3.788	0.302	−11.177	3.601	0.268	1.659	0.335	−1.799	5.117
	5	0.232	0.163	0.730	−0.814	1.140	0.545	−3.188	0.386	−10.646	4.271	0.303	1.746	0.339	−1.941	5.432
Chloride (mg)	1	0.025	0.302	0.399	−0.421	1.025	0.052	−2.620	0.187	−6.575	1.335	0.068	1.224	0.119	−0.331	2.780
	2	0.164	0.410	0.472	−0.745	1.565	0.091	−5.102	0.181	−12.716	2.513	0.209	−0.488	0.728	−3.329	2.352
	3	0.270	0.771	0.188	−0.402	1.945	0.129	−5.068	0.182	−12.658	2.521	0.210	−0.467	0.745	−3.364	2.431
	4	0.280	0.854	0.180	−0.424	2.132	0.340	−6.499	0.070	−13.578	0.579	0.247	−0.497	0.735	−3.465	2.471
	5	0.295	0.905	0.192	−0.497	2.308	0.553	−3.676	0.284	−10.607	3.256	0.283	−0.630	0.680	−3.732	2.471
Copper (mg)	1	0.022	−0.242	0.439	−0.875	0.390	0.032	−2.426	0.308	−7.187	2.336	0.025	0.677	0.347	−0.766	2.119
	2	0.277	−1.073	** 0.044 **	−2.113	−0.033	0.050	−2.194	0.480	−8.463	4.074	0.249	−1.382	0.187	−3.469	0.706
	3	0.320	−0.977	0.065	−2.021	0.066	0.079	−1.322	0.683	−7.884	5.241	0.249	−1.380	0.200	−3.530	0.770
	4	0.329	−1.084	0.069	−2.262	0.093	0.264	−1.868	0.537	−7.989	4.254	0.292	−1.577	0.173	−3.883	0.730
	5	0.416	−1.901	** 0.023 **	−3.508	−0.294	0.535	−1.471	0.585	−6.964	4.023	0.333	−1.754	0.154	−4.208	0.699
Iodine (μg)	1	0.008	0.154	0.637	−0.508	0.816	0.046	−3.572	0.214	−9.309	2.165	0.058	1.392	0.151	−0.533	3.316
	2	0.147	0.073	0.856	−0.749	0.895	0.061	−3.252	0.361	−10.412	3.909	0.219	0.838	0.464	−1.468	3.144
	3	0.219	0.147	0.711	−0.664	0.958	0.084	−2.203	0.559	−9.821	5.415	0.231	1.288	0.337	−1.405	3.980
	4	0.223	0.176	0.681	−0.701	1.054	0.274	−3.022	0.391	−10.129	4.084	0.254	0.919	0.526	−2.011	3.850
	5	0.230	0.118	0.811	−0.902	1.138	0.562	−3.979	0.200	−10.211	2.254	0.280	0.392	0.807	−2.878	3.663
Iron (mg)	1	0.004	−0.110	0.745	−0.793	0.574	0.070	−3.652	0.126	−8.384	1.079	0.031	0.843	0.294	−0.763	2.448
	2	0.186	−0.536	0.276	−1.527	0.456	0.130	−7.646	0.079	−16.241	0.950	0.232	−1.264	0.302	−3.721	1.193
	3	0.240	−0.430	0.381	−1.425	0.565	0.162	−7.387	0.090	−15.990	1.216	0.234	−1.268	0.308	−3.766	1.229
	4	0.254	−0.564	0.307	−1.683	0.555	0.296	−5.258	0.211	−13.683	3.166	0.265	−1.186	0.369	−3.842	1.470
	5	0.280	−0.830	0.254	−2.308	0.647	0.533	−1.636	0.675	−9.604	6.332	0.300	−1.215	0.377	−3.992	1.562
Magnesium (mg)	1	0.060	−0.533	0.193	−1.350	0.285	0.031	−2.638	0.316	−7.904	2.629	0.032	1.051	0.288	−0.927	3.030
	2	0.358	−1.611	** 0.008 **	−2.764	−0.457	0.056	−3.446	0.411	−11.880	4.988	0.249	−2.229	0.184	−5.574	1.116
	3	0.370	−1.470	** 0.023 **	−2.717	−0.224	0.094	−3.321	0.426	−11.741	5.099	0.249	−2.254	0.197	−5.739	1.230
	4	0.376	−1.485	** 0.027 **	−2.790	−0.181	0.274	−3.379	0.385	−11.234	4.477	0.284	−2.378	0.214	−6.208	1.453
	5	0.402	−1.658	** 0.030 **	−3.132	−0.183	0.541	−2.652	0.457	−9.899	4.595	0.315	−2.340	0.245	−6.386	1.707
Manganese (mg)	1	0.036	−0.313	0.315	−0.939	0.314	0.004	0.612	0.724	−2.888	4.111	0.000	0.020	0.978	−1.440	1.479
	2	0.216	−0.670	0.148	−1.595	0.255	0.070	2.447	0.286	−2.151	7.044	0.318	−2.065	** 0.029 **	−3.905	−0.225
	3	0.259	−0.548	0.243	−1.492	0.397	0.110	2.437	0.286	−2.147	7.020	0.319	−2.116	** 0.031 **	−4.029	−0.202
	4	0.261	−0.603	0.268	−1.702	0.496	0.258	0.917	0.683	−3.649	5.484	0.374	−2.394	** 0.020 **	−4.386	−0.403
	5	0.300	−1.023	0.177	−2.551	0.505	0.531	0.672	0.751	−3.650	4.994	0.403	−2.436	** 0.027 **	−4.579	−0.294
Nitrogen (g)	1	0.000	0.029	0.951	−0.925	0.983	0.079	−4.872	0.102	−10.761	1.017	0.069	1.529	0.116	−0.396	3.455
	2	0.159	−0.492	0.540	−2.124	1.140	0.151	−9.958	0.051	−19.945	0.028	0.207	0.307	0.849	−2.949	3.564
	3	0.217	−0.232	0.775	−1.892	1.428	0.182	−9.612	0.059	−19.620	0.395	0.209	0.363	0.826	−2.979	3.706
	4	0.219	−0.201	0.816	−1.965	1.564	0.290	−6.283	0.249	−17.236	4.670	0.244	0.055	0.976	−3.551	3.660
	5	0.227	−0.073	0.941	−2.088	1.943	0.539	−3.647	0.482	−14.194	6.899	0.279	−0.286	0.878	−4.078	3.506
Phosphorous (mg)	1	0.002	0.113	0.809	−0.834	1.059	0.040	−3.794	0.252	−10.412	2.823	0.079	1.913	0.091	−0.325	4.150
	2	0.150	−0.317	0.743	−2.289	1.654	0.080	−7.676	0.228	−20.427	5.075	0.206	0.152	0.954	−5.234	5.538
	3	0.214	−0.017	0.986	−2.004	1.971	0.113	−7.102	0.266	−19.910	5.705	0.208	0.352	0.901	−5.375	6.080
	4	0.217	0.019	0.985	−2.116	2.155	0.279	−5.937	0.332	−18.279	6.405	0.244	0.307	0.922	−6.092	6.706
	5	0.227	0.046	0.968	−2.321	2.414	0.544	−5.139	0.396	−17.430	7.152	0.278	−0.356	0.917	−7.329	6.617
Potassium (mg)	1	0.093	−0.631	0.101	−1.395	0.132	0.041	−3.221	0.244	−8.742	2.301	0.034	1.259	0.276	−1.051	3.569
	2	0.343	−1.289	** 0.011 **	−2.260	−0.318	0.074	−5.037	0.265	−14.094	4.021	0.249	−2.497	0.187	−6.267	1.273
	3	0.359	−1.165	** 0.029 **	−2.199	−0.131	0.103	−4.377	0.338	−13.563	4.810	0.249	−2.577	0.197	−6.564	1.410
	4	0.369	−1.203	** 0.031 **	−2.289	−0.117	0.292	−5.001	0.238	−13.501	3.499	0.278	−2.493	0.252	−6.853	1.866
	5	0.390	−1.305	** 0.036 **	−2.518	−0.092	0.571	−5.495	0.147	−13.061	2.070	0.308	−2.391	0.301	−7.047	2.265
Selenium (μg)	1	0.001	0.070	0.844	−0.649	0.788	0.034	−2.930	0.290	−8.476	2.616	0.088	1.241	0.074	−0.127	2.609
	2	0.154	−0.296	0.630	−1.548	0.955	0.050	−2.893	0.486	−11.263	5.476	0.212	0.502	0.631	−1.608	2.611
	3	0.219	−0.223	0.713	−1.458	1.012	0.076	−1.390	0.756	−10.438	7.658	0.214	0.550	0.609	−1.619	2.720
	4	0.221	−0.210	0.741	−1.513	1.092	0.254	−0.467	0.921	−10.095	9.161	0.246	0.352	0.749	−1.882	2.586
	5	0.231	−0.221	0.747	−1.638	1.195	0.548	−4.096	0.336	−12.718	4.526	0.278	0.059	0.960	−2.358	2.475
Sodium (mg)	1	0.026	0.298	0.396	−0.410	1.006	0.043	−2.386	0.233	−6.379	1.606	0.070	1.203	0.114	−0.303	2.709
	2	0.167	0.429	0.440	−0.696	1.553	0.071	−4.127	0.279	−11.770	3.516	0.208	−0.339	0.802	−3.072	2.394
	3	0.269	0.733	0.193	−0.397	1.864	0.110	−4.098	0.280	−11.719	3.522	0.209	−0.327	0.812	−3.108	2.454
	4	0.278	0.805	0.186	−0.417	2.026	0.316	−5.590	0.128	−12.890	1.711	0.245	−0.350	0.804	−3.209	2.510
	5	0.303	0.938	0.166	−0.426	2.302	0.537	−2.196	0.541	−9.513	5.121	0.282	−0.535	0.716	−3.521	2.451
Zinc (mg)	1	0.002	0.104	0.811	−0.777	0.985	0.037	−3.221	0.270	−9.062	2.621	0.092	1.805	0.068	−0.144	3.754
	2	0.151	−0.285	0.705	−1.818	1.247	0.084	−5.927	0.208	−15.327	3.474	0.210	0.639	0.691	−2.604	3.881
	3	0.215	−0.045	0.952	−1.594	1.504	0.141	−6.936	0.142	−16.332	2.459	0.211	0.653	0.689	−2.645	3.951
	4	0.217	−0.027	0.973	−1.659	1.605	0.272	−3.872	0.410	−13.369	5.625	0.245	0.416	0.806	−3.019	3.851
	5	0.229	0.244	0.820	−1.971	2.459	0.530	0.535	0.908	−8.905	9.975	0.279	0.273	0.877	−3.320	3.866

Abbreviations: NODAP = New-onset diabetes or prediabetes after acute pancreatitis. T2DM = Type 2 diabetes or prediabetes prior to acute pancreatitis. NAP = Normoglycaemia after acute pancreatitis. 95% CI = 95% confidence interval. Footnotes: Data are presented as R-squared values (from crude analysis), unstandardised B, *p* values (from linear regression) and 95% confidence intervals. All the variables were log-transformed. Model 1: unadjusted model. Model 2: age, sex, daily energy intake. Model 3: age, sex, daily energy intake, V/S fat volume ratio. Model 4: age, sex, daily energy intake, V/S fat volume ratio, alcohol intake, smoking status. Model 5: age, sex, daily energy intake, V/S fat volume ratio, alcohol intake, smoking status, aetiology of AP, number of AP episodes, cholecystectomy, use of antidiabetic medications. Significance was set at *p* < 0.05. Significant values are shown in bold.

**Table 5:** Associations between habitual mineral intake and fasting insulin in the study groups.

Mineral	Model	NAP					T2DM					NODAP				
		R^2^	Unstandardised	*p*	95% CI		R^2^	Unstandardised	*p*	95% CI		R^2^	Unstandardised	*p*	95% CI	
			B		Lower	Upper		B		Lower	Upper		B		Lower	Upper
Calcium (mg)	1	0.000	0.682	0.944	−18.900	20.265	0.000	3.095	0.966	−144.004	150.194	0.025	11.785	0.352	−13.594	37.164
	2	0.003	1.530	0.908	−25.514	28.575	0.093	94.086	0.370	−117.360	305.531	0.140	0.416	0.982	−36.564	37.396
	3	0.006	1.318	0.923	−26.345	28.981	0.095	94.223	0.378	−121.278	309.724	0.154	4.158	0.828	−34.648	42.963
	4	0.055	−1.750	0.905	−31.605	28.104	0.345	151.133	0.123	−43.802	346.068	0.233	4.652	0.806	−33.719	43.023
	5	0.169	−4.304	0.779	−35.829	27.222	0.554	211.928	** 0.048 **	2.380	421.476	0.305	3.631	0.853	−36.272	43.535
Chloride (mg)	1	0.000	−0.396	0.971	−22.804	22.013	0.125	−115.711	** 0.043 **	−227.763	−3.659	0.111	16.935	** 0.044 **	0.477	33.394
	2	0.003	−1.419	0.940	−39.663	36.826	0.205	−250.464	** 0.035 **	−482.368	−18.559	0.165	15.112	0.338	−16.512	46.736
	3	0.006	−3.765	0.856	−46.016	38.486	0.206	−249.783	** 0.039 **	−486.616	−12.949	0.181	16.096	0.312	−15.873	48.066
	4	0.054	0.348	0.987	−44.621	45.316	0.325	−157.937	0.202	−405.953	90.079	0.248	12.594	0.430	−19.553	44.740
	5	0.167	3.869	0.865	−42.829	50.567	0.468	−74.265	0.573	−344.241	195.710	0.316	10.893	0.501	−21.922	43.708
Copper (mg)	1	0.001	−1.361	0.890	−21.340	18.619	0.051	−77.657	0.206	−200.404	45.090	0.015	5.743	0.463	−9.966	21.452
	2	0.005	−4.421	0.814	−42.569	33.727	0.087	−62.201	0.431	−221.550	97.148	0.175	−13.480	0.255	−37.185	10.225
	3	0.008	−5.274	0.785	−44.670	34.123	0.094	−73.003	0.383	−242.022	96.016	0.182	−12.505	0.302	−36.818	11.809
	4	0.057	−6.079	0.775	−49.572	37.413	0.309	−78.894	0.304	−233.561	75.772	0.239	−7.103	0.579	−33.004	18.797
	5	0.166	−0.005	1.000	−58.549	58.538	0.469	−43.971	0.561	−198.733	110.791	0.313	−7.846	0.555	−34.827	19.134
Iodine (μg)	1	0.015	6.774	0.508	−13.910	27.458	0.011	44.089	0.559	−108.095	196.274	0.003	−3.376	0.751	−24.832	18.080
	2	0.028	10.929	0.421	−16.526	38.385	0.145	139.849	0.119	−38.148	317.846	0.183	−16.155	0.207	−41.723	9.412
	3	0.029	10.675	0.444	−17.574	38.924	0.149	149.525	0.121	−42.249	341.299	0.183	−15.662	0.296	−45.737	14.413
	4	0.064	7.312	0.619	−22.663	37.287	0.391	177.429	** 0.042 **	7.071	347.788	0.262	−17.142	0.276	−48.728	14.444
	5	0.167	2.035	0.898	−30.667	34.737	0.560	173.350	** 0.041 **	7.943	338.758	0.389	−30.162	0.069	−62.806	2.482
Iron (mg)	1	0.003	3.297	0.755	−18.119	24.712	0.099	−115.820	0.075	−243.956	12.315	0.065	13.101	0.129	−3.999	30.202
	2	0.011	7.683	0.648	−26.530	41.897	0.160	−200.167	0.088	−432.286	31.952	0.141	1.804	0.897	−26.366	29.974
	3	0.012	7.195	0.679	−28.208	42.597	0.162	−201.193	0.092	−437.755	35.369	0.153	1.665	0.906	−26.784	30.113
	4	0.066	10.265	0.592	−28.802	49.333	0.407	−245.327	** 0.028 **	−462.181	−28.472	0.242	9.479	0.512	−19.738	38.695
	5	0.219	26.432	0.260	−21.085	73.948	0.506	−162.179	0.177	−403.620	79.263	0.316	9.995	0.496	−19.730	39.721
Magnesium (mg)	1	0.004	4.215	0.745	−22.047	30.478	0.031	−69.528	0.325	−211.363	72.307	0.064	16.067	0.131	−5.016	37.150
	2	0.013	11.065	0.615	−33.550	55.680	0.070	−38.358	0.729	−262.673	185.957	0.142	4.207	0.826	−34.552	42.967
	3	0.013	10.442	0.663	−38.328	59.212	0.072	−38.529	0.732	−267.158	190.100	0.157	7.364	0.710	−32.652	47.381
	4	0.064	11.718	0.631	−38.103	61.538	0.279	−13.693	0.895	−225.423	198.038	0.264	23.641	0.260	−18.415	65.697
	5	0.178	13.735	0.599	−39.821	67.291	0.467	56.533	0.604	−166.590	279.656	0.346	27.072	0.205	−15.765	69.909
Manganese (mg)	1	0.011	5.508	0.569	−14.050	25.066	0.041	−51.721	0.256	−142.781	39.339	0.021	6.635	0.395	−9.016	22.286
	2	0.027	12.556	0.426	−19.325	44.437	0.074	−29.026	0.632	−151.707	93.654	0.145	−4.331	0.695	−26.653	17.990
	3	0.027	12.358	0.457	−21.338	46.054	0.076	−28.812	0.640	−153.877	96.254	0.155	−2.854	0.803	−25.954	20.246
	4	0.063	8.552	0.651	−30.036	47.141	0.282	−21.648	0.713	−141.639	98.343	0.232	1.811	0.878	−22.102	25.723
	5	0.191	18.914	0.446	−31.778	69.606	0.464	23.069	0.710	−104.147	150.285	0.308	4.983	0.685	−20.009	29.976
Nitrogen (g)	1	0.006	6.296	0.667	−23.334	35.925	0.141	−164.493	** 0.032 **	−313.440	−15.546	0.023	9.547	0.371	−11.829	30.923
	2	0.026	20.629	0.436	−32.961	74.219	0.220	−290.208	** 0.026 **	−543.873	−36.544	0.149	−10.206	0.574	−46.762	26.350
	3	0.026	20.417	0.469	−36.755	77.589	0.224	−293.149	** 0.028 **	−551.738	−34.560	0.159	−8.631	0.641	−45.969	28.707
	4	0.067	16.507	0.572	−43.109	76.122	0.449	−354.028	** 0.010 **	−616.299	−91.757	0.231	−2.819	0.885	−42.203	36.565
	5	0.205	29.712	0.336	−33.177	92.601	0.557	−302.235	** 0.043 **	−594.534	−9.936	0.309	−8.424	0.670	−48.642	31.795
Phosphorous (mg)	1	0.005	5.317	0.715	−24.217	34.852	0.064	−124.221	0.154	−297.555	49.113	0.030	12.826	0.303	−12.057	37.708
	2	0.028	26.005	0.417	−38.750	90.760	0.104	−176.359	0.291	−511.914	159.195	0.154	−21.636	0.470	−81.865	38.594
	3	0.028	25.670	0.448	−42.882	94.222	0.106	−180.110	0.290	−522.839	162.618	0.161	−17.082	0.589	−80.946	46.783
	4	0.065	17.927	0.614	−54.521	90.374	0.294	−120.851	0.458	−451.367	209.664	0.231	2.698	0.938	−67.235	72.630
	5	0.181	21.602	0.558	−53.968	97.173	0.461	−38.232	0.840	−427.352	350.887	0.306	2.698	0.938	−67.235	72.630
Potassium (mg)	1	0.006	5.082	0.680	−19.831	29.995	0.004	−25.770	0.734	−179.222	127.681	0.038	14.536	0.245	−10.432	39.504
	2	0.014	9.796	0.592	−27.342	46.933	0.077	67.976	0.576	−177.997	313.950	0.147	−10.519	0.626	−54.057	33.018
	3	0.014	9.292	0.637	−30.771	49.355	0.078	65.721	0.599	−187.360	318.802	0.156	−6.998	0.758	−52.818	38.823
	4	0.063	9.372	0.643	−31.888	50.632	0.294	83.250	0.466	−148.104	314.604	0.234	8.031	0.738	−40.624	56.686
	5	0.177	10.860	0.609	−32.768	54.489	0.486	115.795	0.314	−117.857	349.446	0.312	13.207	0.594	−37.094	63.508
Selenium (μg)	1	0.009	5.761	0.602	−16.608	28.130	0.098	−125.002	0.076	−263.713	13.708	0.057	10.798	0.155	−4.281	25.877
	2	0.034	18.588	0.369	−23.220	60.395	0.114	−127.226	0.229	−339.267	84.815	0.144	4.334	0.713	−19.488	28.156
	3	0.035	18.255	0.389	−24.650	61.159	0.132	−160.099	0.169	−392.384	72.187	0.159	5.725	0.634	−18.588	30.037
	4	0.076	15.940	0.464	−28.303	60.183	0.287	−66.978	0.582	−314.052	180.097	0.236	5.128	0.670	−19.252	29.508
	5	0.187	15.431	0.485	−29.824	60.687	0.489	−126.832	0.291	−370.420	116.757	0.304	0.815	0.949	−24.893	26.523
Sodium (mg)	1	0.001	−1.545	0.887	−23.518	20.428	0.159	−130.487	** 0.022 **	−240.412	−20.562	0.112	16.517	**0.043**	0.577	32.457
	2	0.005	−4.201	0.818	−41.446	33.043	0.275	−299.924	** 0.008 **	−516.198	−83.650	0.167	15.217	0.315	−15.142	45.576
	3	0.010	−6.605	0.740	−47.140	33.930	0.276	−299.407	** 0.010 **	−520.277	−78.537	0.182	15.680	0.305	−14.964	46.323
	4	0.056	−4.007	0.848	−46.888	38.874	0.359	−212.348	0.089	−459.386	34.690	0.247	11.938	0.437	−19.015	42.891
	5	0.167	2.849	0.898	−42.868	48.566	0.489	−142.541	0.288	−414.470	129.388	0.314	9.383	0.547	−22.242	41.008
Zinc (mg)	1	0.004	4.532	0.737	−22.804	31.869	0.213	−197.213	** 0.007 **	−336.116	−58.310	0.035	12.018	0.270	−9.755	33.791
	2	0.018	15.253	0.535	−34.560	65.067	0.382	−373.530	** 0.001 **	−575.644	−171.417	0.143	−5.245	0.772	−41.851	31.362
	3	0.018	14.702	0.573	−38.378	67.781	0.386	−380.543	** 0.001 **	−589.288	−171.797	0.155	−4.778	0.794	−41.776	32.220
	4	0.066	14.441	0.590	−40.152	69.033	0.657	−445.092	** <0.001 **	−619.351	−270.832	0.231	−1.635	0.930	−39.212	35.941
	5	0.260	50.565	0.128	−15.779	116.909	0.724	−443.991	** <0.001 **	−650.255	−237.727	0.305	−4.170	0.824	−42.378	34.037

Abbreviations: NODAP = New-onset diabetes or prediabetes after acute pancreatitis. T2DM = Type 2 diabetes or prediabetes prior to acute pancreatitis. NAP = Normoglycaemia after acute pancreatitis. 95% CI = 95% confidence interval. Footnotes: Data are presented as R-squared values (from crude analysis), unstandardised B, *p* values (from linear regression) and 95% confidence intervals. All the variables were log-transformed. Model 1: unadjusted model. Model 2: age, sex, daily energy intake. Model 3: age, sex, daily energy intake, V/S fat volume ratio. Model 4: age, sex, daily energy intake, V/S fat volume ratio, alcohol intake, smoking status. Model 5: age, sex, daily energy intake, V/S fat volume ratio, alcohol intake, smoking status, aetiology of AP, number of AP episodes, cholecystectomy, use of antidiabetic medications. Significance was set at *p* < 0.05. Significant values are shown in bold.

**Table 6:** Associations between habitual mineral intake and HOMA-S in the study groups.

Mineral	Model	NAP					T2DM					NODAP				
		R^2^	Unstandardised	*p*	95% CI		R^2^	Unstandardised	*p*	95% CI		R^2^	Unstandardised	*p*	95% CI	
			B		Lower	Upper		B		Lower	Upper		B		Lower	Upper
Calcium (mg)	1	0.000	4.245	0.926	−88.813	97.302	0.001	−8.964	0.867	−117.484	99.556	0.001	−20.276	0.881	−293.772	253.221
	2	0.073	23.013	0.706	−101.166	147.192	0.067	−18.272	0.817	−179.031	142.487	0.107	114.173	0.563	−283.789	512.134
	3	0.073	22.983	0.713	−104.289	150.254	0.192	−16.230	0.829	−168.916	136.457	0.110	132.410	0.526	−288.485	553.304
	4	0.154	41.110	0.533	−93.534	175.754	0.223	−5.025	0.950	−169.995	159.946	0.229	139.137	0.489	−267.284	545.558
	5	0.247	66.633	0.345	−77.518	210.784	0.420	−88.801	0.348	−281.674	104.072	0.709	57.731	0.667	−215.061	330.524
Chloride (mg)	1	0.033	−51.011	0.339	−158.443	56.421	0.004	15.765	0.737	−79.199	110.730	0.111	−180.055	**0.044**	−355.117	−4.993
	2	0.086	−63.180	0.477	−243.526	117.166	0.086	77.326	0.432	−121.453	276.104	0.178	−289.538	0.086	−622.669	43.594
	3	0.089	−72.394	0.452	−268.015	123.228	0.216	82.758	0.374	−105.362	270.879	0.178	−289.377	0.092	−629.320	50.567
	4	0.181	−104.669	0.298	−308.129	98.790	0.264	111.304	0.256	−86.304	308.911	0.280	−261.122	0.121	−594.969	72.724
	5	0.246	−98.220	0.355	−315.089	118.648	0.400	49.419	0.637	−165.851	264.689	0.750	−217.292	**0.044**	−428.069	−6.514
Copper (mg)	1	0.031	−43.362	0.352	−137.263	50.538	0.009	24.564	0.599	−69.756	118.884	0.099	−154.495	0.057	−314.111	5.122
	2	0.092	−69.511	0.416	−242.574	103.551	0.102	60.255	0.299	−56.496	177.005	0.146	−170.409	0.186	−427.502	86.684
	3	0.093	−71.292	0.418	−249.976	107.392	0.199	30.092	0.604	−87.746	147.929	0.146	−171.753	0.196	−436.630	93.124
	4	0.165	−78.492	0.413	−273.392	116.408	0.228	22.564	0.707	−99.655	144.782	0.309	−253.252	0.058	−515.926	9.422
	5	0.212	−31.411	0.815	−308.032	245.211	0.283	10.249	0.871	−119.765	140.263	0.745	−167.033	0.061	−342.373	8.307
Iodine (μg)	1	0.004	−15.292	0.755	−114.575	83.990	0.010	30.916	0.579	−81.679	143.511	0.020	−94.323	0.404	−320.836	132.190
	2	0.067	4.789	0.939	−123.720	133.298	0.087	56.379	0.421	−85.043	197.802	0.108	−85.555	0.543	−368.826	197.716
	3	0.067	4.607	0.943	−127.926	137.140	0.192	14.082	0.842	−129.786	157.951	0.109	−100.274	0.542	−432.233	231.685
	4	0.146	28.682	0.670	−109.020	166.384	0.226	24.852	0.743	−130.091	179.795	0.220	−62.038	0.715	−405.720	281.644
	5	0.238	60.435	0.415	−91.349	212.218	0.282	3.760	0.964	−165.192	172.712	0.730	166.340	0.149	−63.802	396.482
Iron (mg)	1	0.051	−59.579	0.231	−159.174	40.016	0.000	−1.752	0.973	−107.430	103.927	0.115	−185.833	**0.040**	−363.132	−8.535
	2	0.109	−82.280	0.286	−237.781	73.220	0.067	24.843	0.789	−164.093	213.779	0.165	−233.936	0.116	−528.950	61.077
	3	0.111	−85.085	0.287	−246.254	76.083	0.192	14.699	0.868	−165.163	194.562	0.166	−234.282	0.121	−534.284	65.720
	4	0.206	−114.133	0.186	−287.501	59.234	0.226	30.623	0.755	−169.964	231.209	0.352	−346.007	**0.020**	−633.371	−58.643
	5	0.254	−115.084	0.302	−342.302	112.134	0.282	3.634	0.972	−211.245	218.513	0.815	−311.717	**0.001**	−476.081	−147.352
Magnesium (mg)	1	0.003	−19.157	0.758	−145.201	106.887	0.000	−2.214	0.967	−110.488	106.061	0.037	−129.590	0.256	−357.405	98.225
	2	0.068	12.933	0.899	−194.469	220.335	0.065	2.267	0.978	−163.449	167.982	0.102	−83.355	0.690	−505.305	338.596
	3	0.068	13.362	0.904	−212.038	238.763	0.191	0.629	0.994	−156.748	158.006	0.102	−80.624	0.711	−520.302	359.053
	4	0.138	6.492	0.953	−221.286	234.269	0.223	9.332	0.906	−152.865	171.528	0.251	−255.197	0.258	−707.649	197.254
	5	0.217	48.798	0.688	−201.321	298.916	0.288	−38.814	0.670	−225.826	148.199	0.744	−270.454	0.062	−556.016	15.109
Manganese (mg)	1	0.033	−44.415	0.337	−137.646	48.815	0.025	−29.789	0.387	−99.021	39.444	0.018	−64.901	0.434	−231.423	101.621
	2	0.107	−75.452	0.299	−221.969	71.064	0.085	−34.271	0.443	−124.527	55.986	0.097	−8.935	0.941	−252.480	234.611
	3	0.110	−80.190	0.292	−233.884	73.504	0.210	−33.277	0.432	−118.905	52.352	0.098	−5.432	0.965	−259.170	248.305
	4	0.157	−58.269	0.495	−232.216	115.678	0.259	−47.567	0.288	−137.956	42.823	0.219	−38.405	0.761	−294.475	217.665
	5	0.210	0.653	0.996	−240.491	241.797	0.369	−82.329	0.102	−182.399	17.741	0.708	−27.175	0.749	−199.621	145.271
Nitrogen (g)	1	0.038	−71.506	0.304	−211.519	68.508	0.015	42.746	0.499	−84.772	170.264	0.070	−177.381	0.113	−398.803	44.041
	2	0.112	−137.456	0.271	−389.188	114.275	0.111	124.875	0.248	−92.253	342.003	0.146	−258.927	0.184	−647.304	129.451
	3	0.116	−146.926	0.263	−411.659	117.807	0.223	105.339	0.308	−102.865	313.543	0.147	−260.240	0.193	−659.486	139.006
	4	0.174	−127.030	0.343	−399.145	145.084	0.314	213.001	0.087	−33.151	459.154	0.281	−317.497	0.119	−721.219	86.225
	5	0.259	−157.445	0.278	−452.534	137.643	0.370	224.107	0.101	−47.412	495.625	0.732	−202.711	0.129	−468.542	63.121
Phosphorous (mg)	1	0.012	−39.950	0.563	−179.722	99.821	0.004	22.295	0.741	−114.093	158.684	0.029	−133.702	0.311	−397.468	130.064
	2	0.072	−51.373	0.734	−359.027	256.282	0.080	84.240	0.504	−170.846	339.326	0.102	−132.413	0.687	−794.890	530.063
	3	0.072	−54.417	0.729	−375.257	266.423	0.200	65.482	0.585	−178.166	309.130	0.102	−129.617	0.710	−833.766	574.532
	4	0.138	−7.089	0.965	−340.158	325.981	0.243	98.521	0.432	−156.148	353.190	0.241	−353.553	0.340	−1099.203	392.097
	5	0.212	31.093	0.857	−325.047	387.233	0.286	56.721	0.713	−259.758	373.200	0.720	−269.348	0.284	−775.847	237.152
Potassium (mg)	1	0.003	−16.429	0.781	−136.370	103.512	0.004	18.905	0.736	−94.355	132.165	0.027	−128.753	0.334	−395.441	137.936
	2	0.067	3.992	0.962	−168.705	176.690	0.082	64.340	0.479	−119.569	248.249	0.097	−1.281	0.996	−478.077	475.515
	3	0.067	3.486	0.969	−182.688	189.661	0.198	40.995	0.639	−136.289	218.279	0.098	9.198	0.971	−496.937	515.333
	4	0.138	2.516	0.978	−186.768	191.800	0.233	49.116	0.585	−133.876	232.109	0.229	−172.371	0.502	−691.495	346.753
	5	0.215	35.293	0.723	−170.311	240.896	0.282	13.879	0.892	−195.396	223.154	0.723	−206.074	0.222	−544.658	132.510
Selenium (μg)	1	0.082	−79.157	0.126	−181.980	23.665	0.002	−11.955	0.832	−126.209	102.300	0.156	−190.212	**0.015**	−341.928	−38.496
	2	0.174	−161.698	0.084	−346.864	23.467	0.065	−9.716	0.905	−175.647	156.215	0.270	−316.557	**0.010**	−550.548	−82.565
	3	0.175	−163.257	0.089	−353.246	26.733	0.225	−88.172	0.294	−257.424	81.081	0.272	−321.541	**0.010**	−562.267	−80.815
	4	0.229	−150.724	0.121	−344.671	43.224	0.239	−65.983	0.483	−256.883	124.918	0.365	−301.402	**0.014**	−538.260	−64.544
	5	0.284	−136.762	0.178	−341.488	67.963	0.287	−41.705	0.682	−250.337	166.927	0.751	−170.803	**0.042**	−334.944	−6.663
Sodium (mg)	1	0.045	−58.808	0.260	−163.574	45.959	0.004	16.363	0.733	−80.536	113.262	0.124	−184.187	**0.033**	−352.486	−15.889
	2	0.106	−88.626	0.307	−263.553	86.301	0.091	87.005	0.384	−114.584	288.594	0.199	−311.853	0.052	−626.458	2.752
	3	0.111	−98.167	0.289	−285.097	88.764	0.221	93.598	0.322	−97.008	284.204	0.199	−311.400	0.056	−631.659	8.858
	4	0.203	−124.509	0.194	−317.339	68.320	0.281	137.051	0.176	−65.969	340.071	0.298	−283.647	0.077	−599.697	32.403
	5	0.271	−126.663	0.223	−336.940	83.615	0.343	144.558	0.175	−69.341	358.456	0.754	−218.425	**0.033**	−418.330	−18.520
Zinc (mg)	1	0.023	−51.834	0.420	−181.514	77.846	0.025	55.468	0.385	−72.923	183.859	0.073	−183.978	0.105	−408.329	40.372
	2	0.085	−78.844	0.495	−313.081	155.394	0.112	125.731	0.243	−90.458	341.919	0.145	−252.328	0.191	−637.361	132.704
	3	0.086	−83.949	0.488	−329.674	161.776	0.273	169.479	0.098	−33.648	372.607	0.145	−251.641	0.200	−643.524	140.241
	4	0.158	−86.306	0.480	−335.522	162.909	0.377	289.421	**0.023**	43.866	534.977	0.264	−258.584	0.182	−645.443	128.275
	5	0.229	−105.373	0.507	−431.429	220.683	0.419	309.600	**0.037**	20.715	598.485	0.742	−224.960	0.070	−469.860	19.940

Abbreviations: NODAP = New-onset diabetes or prediabetes after acute pancreatitis. T2DM = Type 2 diabetes or prediabetes prior to acute pancreatitis. NAP = Normoglycaemia after acute pancreatitis. HOMA-S = homeostasis model assessment of insulin sensitivity. Footnotes: Data are presented as R-squared values (from crude analysis), unstandardised B, *p* values (from linear regression) and 95% confidence intervals. All the variables were log-transformed. Model 1: unadjusted model. Model 2: age, sex, daily energy intake. Model 3: age, sex, daily energy intake, V/S fat volume ratio. Model 4: age, sex, daily energy intake, V/S fat volume ratio, alcohol intake, smoking status. Model 5: age, sex, daily energy intake, V/S fat volume ratio, alcohol intake, smoking status, aetiology of AP, number of AP episodes, cholecystectomy, use of antidiabetic medications. Significance was set at *p* < 0.05. Significant values are shown in bold.

**Table 7:** Associations between habitual mineral intake and HOMA-β in the study groups.

Mineral	Model	NAP					T2DM					NODAP				
		R^2^	Unstandardised	*p*	95% CI		R^2^	Unstandardised	*p*	95% CI		R^2^	Unstandardised	*p*	95% CI	
			B		Lower	Upper		B		Lower	Upper		B		Lower	Upper
Calcium (mg)	1	0.000	−5.939	0.924	−131.853	119.975	0.026	59.501	0.375	−75.352	194.355	0.002	−14.743	0.801	−132.376	102.890
	2	0.012	−6.229	0.942	−179.720	167.261	0.073	77.512	0.436	−123.813	278.838	0.045	−38.284	0.665	−216.884	140.317
	3	0.026	−9.389	0.914	−185.937	167.158	0.082	78.258	0.439	−126.297	282.813	0.045	−36.058	0.700	−224.993	152.877
	4	0.096	−29.370	0.750	−218.038	159.298	0.199	100.850	0.333	−109.921	311.621	0.122	−31.336	0.736	−219.349	156.676
	5	0.207	−49.778	0.609	−249.916	150.361	0.323	212.290	0.108	−51.282	475.862	0.242	−36.731	0.696	−227.639	154.177
Chloride (mg)	1	0.000	−6.794	0.926	−154.426	140.838	0.015	39.754	0.501	−79.372	158.881	0.054	54.184	0.166	−23.594	131.962
	2	0.012	−3.849	0.975	−257.061	249.363	0.062	68.869	0.583	−185.206	322.944	0.143	141.793	0.058	−5.019	288.606
	3	0.028	−31.997	0.811	−305.099	241.105	0.071	71.013	0.577	−187.234	329.259	0.147	144.009	0.058	−5.501	293.518
	4	0.092	−3.160	0.982	−292.529	286.208	0.174	61.822	0.633	−201.737	325.382	0.201	128.137	0.095	−23.649	279.924
	5	0.197	17.586	0.904	−284.210	319.383	0.239	81.685	0.583	−223.503	386.873	0.304	116.500	0.126	−35.158	268.159
Copper (mg)	1	0.001	7.765	0.903	−121.090	136.620	0.017	41.359	0.482	−77.394	160.112	0.004	14.096	0.695	−58.279	86.471
	2	0.016	34.751	0.772	−209.890	279.392	0.053	17.738	0.812	−134.068	169.543	0.039	6.490	0.911	−110.693	123.673
	3	0.027	24.732	0.841	−226.887	276.350	0.060	7.625	0.923	−153.952	169.202	0.041	8.557	0.886	−112.062	129.176
	4	0.093	19.065	0.887	−256.912	295.043	0.168	16.570	0.833	−143.875	177.016	0.136	46.493	0.458	−79.998	172.984
	5	0.201	64.828	0.724	−314.034	443.690	0.228	11.032	0.897	−164.469	186.533	0.251	42.761	0.502	−86.363	171.884
Iodine (μg)	1	0.008	30.782	0.640	−102.706	164.271	0.046	81.841	0.240	−57.529	221.212	0.022	−42.133	0.386	−139.513	55.247
	2	0.029	56.302	0.518	−120.504	233.109	0.098	101.984	0.249	−75.679	279.648	0.072	−64.920	0.297	−189.764	59.923
	3	0.039	50.674	0.570	−130.679	232.027	0.098	98.086	0.303	−93.617	289.789	0.076	−78.239	0.285	−224.781	68.303
	4	0.095	23.215	0.803	−167.908	214.337	0.225	127.661	0.189	−67.401	322.723	0.141	−66.715	0.389	−222.844	89.415
	5	0.197	−15.199	0.881	−225.146	194.748	0.309	158.222	0.140	−56.457	372.901	0.322	−137.836	0.084	−295.371	19.699
Iron (mg)	1	0.007	30.044	0.658	−107.487	167.575	0.035	66.164	0.308	−64.121	196.449	0.035	44.412	0.265	−35.151	123.976
	2	0.034	79.261	0.461	−138.613	297.135	0.076	98.022	0.403	−138.853	334.898	0.079	77.703	0.245	−55.889	211.295
	3	0.043	70.665	0.523	−154.431	295.761	0.083	95.313	0.424	−145.887	336.513	0.081	77.519	0.253	−58.319	213.357
	4	0.123	104.201	0.387	−140.508	348.909	0.168	30.333	0.813	−231.155	291.821	0.202	117.228	0.091	−20.114	254.570
	5	0.276	206.316	0.165	−92.421	505.054	0.230	35.933	0.801	−258.075	329.941	0.323	119.810	0.081	−15.689	255.309
Magnesium (mg)	1	0.012	47.893	0.566	−121.097	216.883	0.010	37.288	0.578	−98.261	172.836	0.018	39.442	0.424	−59.480	138.364
	2	0.048	134.307	0.338	−148.960	417.574	0.054	27.146	0.794	−183.772	238.064	0.074	100.091	0.277	−84.314	284.497
	3	0.051	120.287	0.429	−188.080	428.655	0.062	26.630	0.800	−187.738	240.998	0.080	109.211	0.254	−82.253	300.675
	4	0.122	130.433	0.395	−181.386	442.253	0.167	16.960	0.870	−195.391	229.310	0.228	195.903	0.052	−1.449	393.255
	5	0.225	137.590	0.405	−200.651	475.831	0.231	34.706	0.778	−219.204	288.617	0.359	209.968	**0.035**	15.617	404.319
Manganese (mg)	1	0.028	55.427	0.378	−71.380	182.235	0.005	16.415	0.707	−71.921	104.751	0.010	21.010	0.558	−51.092	93.113
	2	0.071	123.704	0.221	−79.364	326.773	0.052	−3.069	0.957	−119.040	112.901	0.050	32.472	0.544	−75.283	140.227
	3	0.075	116.172	0.271	−96.576	328.920	0.060	−2.711	0.963	−120.579	115.156	0.054	36.441	0.512	−75.538	148.420
	4	0.115	87.295	0.463	−155.219	329.809	0.174	26.902	0.649	−93.552	147.355	0.161	67.896	0.235	−46.563	182.354
	5	0.233	148.215	0.348	−174.149	470.580	0.228	6.949	0.921	−136.888	150.786	0.301	86.001	0.137	−29.129	201.130
Nitrogen (g)	1	0.009	45.855	0.628	−145.675	237.384	0.028	72.596	0.361	−87.307	232.498	0.001	10.020	0.838	−89.000	109.040
	2	0.059	187.715	0.277	−160.518	535.947	0.079	122.556	0.377	−157.318	402.431	0.039	−2.024	0.982	−179.990	175.943
	3	0.063	173.300	0.338	−192.896	539.496	0.085	117.196	0.407	−168.841	403.233	0.040	0.506	0.996	−182.241	183.252
	4	0.117	144.646	0.436	−233.597	522.889	0.167	−23.512	0.888	−365.757	318.734	0.127	48.643	0.609	−143.703	240.989
	5	0.253	229.907	0.243	−169.685	629.500	0.228	−24.832	0.898	−421.984	372.320	0.239	19.193	0.840	−174.203	212.590
Phosphorous (mg)	1	0.003	27.993	0.766	−162.724	218.710	0.015	57.456	0.499	−114.202	229.114	0.000	7.146	0.901	−108.600	122.893
	2	0.045	188.086	0.367	−233.295	609.466	0.060	78.072	0.627	−247.944	404.089	0.039	−13.901	0.924	−308.039	280.237
	3	0.051	169.357	0.435	−271.236	609.950	0.067	71.617	0.662	−261.259	404.493	0.040	−5.683	0.971	−318.630	307.265
	4	0.103	113.980	0.613	−346.970	574.930	0.167	17.031	0.918	−321.290	355.353	0.134	117.914	0.484	−222.170	457.998
	5	0.208	126.685	0.590	−356.433	609.803	0.234	85.992	0.685	−349.189	521.173	0.240	54.466	0.755	−300.687	409.619
Potassium (mg)	1	0.018	56.300	0.477	−103.819	216.419	0.006	30.712	0.663	−111.969	173.394	0.008	29.688	0.607	−86.520	145.896
	2	0.047	109.673	0.347	−125.983	345.328	0.051	−1.282	0.991	−235.386	232.821	0.047	51.782	0.620	−159.087	262.650
	3	0.051	97.860	0.434	−155.787	351.506	0.060	−8.590	0.942	−248.425	231.244	0.050	62.325	0.572	−160.321	284.971
	4	0.119	101.462	0.425	−157.261	360.184	0.166	−2.926	0.980	−241.369	235.517	0.161	138.053	0.236	−95.225	371.330
	5	0.222	105.443	0.434	−170.771	381.657	0.228	18.263	0.892	−259.878	296.404	0.293	162.286	0.165	−71.287	395.858
Selenium (μg)	1	0.013	43.158	0.544	−100.802	187.119	0.069	102.133	0.146	−37.525	241.791	0.017	26.818	0.445	−43.726	97.362
	2	0.068	157.873	0.231	−107.040	422.786	0.132	155.801	0.125	−45.927	357.530	0.065	52.255	0.358	−61.844	166.354
	3	0.078	152.266	0.257	−118.360	422.893	0.133	162.882	0.151	−63.501	389.264	0.068	55.403	0.343	−61.864	172.670
	4	0.132	135.082	0.323	−142.104	412.269	0.215	144.228	0.235	−99.935	388.390	0.151	60.494	0.302	−57.238	178.226
	5	0.232	128.958	0.356	−156.548	414.464	0.268	141.726	0.305	−139.159	422.612	0.249	37.843	0.530	−84.499	160.185
Sodium (mg)	1	0.001	−12.310	0.863	−156.880	132.261	0.007	27.329	0.651	−94.818	149.477	0.056	53.368	0.159	−21.949	128.684
	2	0.013	−17.939	0.882	−264.710	228.832	0.051	4.073	0.975	−256.284	264.431	0.145	137.731	0.055	−3.231	278.693
	3	0.030	−43.199	0.737	−305.709	219.310	0.060	5.845	0.964	−258.847	270.537	0.147	138.601	0.058	−4.721	281.923
	4	0.094	−24.835	0.854	−300.837	251.166	0.167	−18.877	0.889	−295.000	257.246	0.199	121.615	0.100	−24.696	267.927
	5	0.196	14.467	0.919	−280.912	309.846	0.228	−20.252	0.897	−341.472	300.969	0.297	105.786	0.150	−40.874	252.447
Zinc (mg)	1	0.003	27.139	0.756	−150.344	204.621	0.002	22.001	0.786	−141.899	185.901	0.002	11.563	0.818	−89.887	113.012
	2	0.038	131.442	0.418	−197.461	460.345	0.051	−2.183	0.987	−284.797	280.430	0.039	6.272	0.943	−171.268	183.813
	3	0.045	115.778	0.494	−228.626	460.181	0.060	9.784	0.946	−281.729	301.297	0.041	6.966	0.938	−173.654	187.585
	4	0.111	115.634	0.501	−235.095	466.363	0.204	−180.999	0.299	−533.080	171.082	0.123	34.254	0.706	−149.606	218.113
	5	0.296	335.168	0.117	−91.712	762.049	0.256	−180.723	0.394	−613.285	251.839	0.239	18.785	0.835	−164.458	202.028

Abbreviations: NODAP = New-onset diabetes or prediabetes after acute pancreatitis. T2DM = Type 2 diabetes or prediabetes prior to acute pancreatitis. NAP = Normoglycaemia after acute pancreatitis. HOMA-B = homeostasis model assessment of b-cell dysfunction. Footnotes: Data are presented as R-squared values (from crude analysis), unstandardised B, *p* values (from linear regression) and 95% confidence intervals. All the variables were log-transformed. Model 1: unadjusted model. Model 2: age, sex, daily energy intake. Model 3: age, sex, daily energy intake, V/S fat volume ratio. Model 4: age, sex, daily energy intake, V/S fat volume ratio, alcohol intake, smoking status. Model 5: age, sex, daily energy intake, V/S fat volume ratio, alcohol intake, smoking status, aetiology of AP, number of AP episodes, cholecystectomy, use of antidiabetic medications. Significance was set at *p* < 0.05. Significant values are shown in bold.

## Data Availability

The data are not publicly available due to the ethical conduct in human research regulations.
